# Integrative molecular mechanisms of lodging resistance in rice (*Oryza sativa* L.) culms: from DELLA–OsGSK2–BZR1 signaling to cell wall engineering and QTL-based breeding

**DOI:** 10.3389/fpls.2026.1922614

**Published:** 2026-10-02

**Authors:** Ahmed Sherif, Cuifeng Tang, Chao Dong, Xinxiang A, Yiding Sun, Yi Yang, Na Ge, Ruimin Nie, Amr A. Hassan, Feifei Zhang, Yayun Yang

**Affiliations:** 1 Biotechnology and Germplasm Resources Institute, Yunnan Academy of Agricultural Sciences, Kunming, Yunnan, China; 2 Rice Research Department, Field Crops Research Institute, Agricultural Research Center, Sakha, Egypt; 3 Yunnan Provincial Key Lab of Agriculture Biotechnology, Kunming, Yunnan, China; 4 Key Lab of Southwestern Crop Gene Resources and Germplasm Innovation, Ministry of Agriculture and Rural Affairs, Kunming, Yunnan, China; 5 Scientific Observation for Rice Germplasm Resources of Yunnan, Ministry of Agriculture and Rural Affairs, Kunming, Yunnan, China; 6 Rice Pathology Department, Plant Pathology Research Institute, Agricultural Research Center, Sakha, Egypt

**Keywords:** cell wall, culm strength, gene editing, lodging resistance, molecular regulation, phytohormones, rice

## Abstract

Rice lodging, permanent culm displacement from the vertical position, reduces grain yield and quality, potentially causing up to ~80% yield loss. Lodging resistance is a multifactorial trait shaped by stem morphology, internal anatomy, and cell wall chemistry. A central unresolved question in the field concerns the relative contribution of lignin versus cellulose to culm mechanical strength, a debate that has significant implications for targeted breeding and cell wall engineering. This review offers a comprehensive integration of two key questions: (1) What are the current molecular regulatory mechanisms underlying lodging resistance-associated culm traits in rice? (2) How can these mechanisms be translated into effective breeding strategies?. How can these mechanisms be translated into effective breeding strategies? Unlike earlier reviews that focused on individual mechanisms, this synthesis integrates hormonal crosstalk, hierarchical NAC-MYB cascades, emerging evidence for epigenetic regulation, and QTL-based breeding into a unified conceptual framework centered on the DELLA-OsGSK2-BZR1 module. Our synthesis reveals that plant architecture is governed by extensive phytohormone crosstalk centered on the proposed DELLA-OsGSK2-BZR1 module, where gibberellin and brassinosteroid synergistically modulate elongation and thickening, whereas auxin, strigolactone, ethylene, and jasmonic acid fine-tune plant height and tillering. Major-effect loci, such as *SCM2/APO1* and *SCM3/OsTB1*, enable the concurrent improvement of strength and yield, with QTL pyramiding reported to increase culm diameter by up to ~39% in specific genetic backgrounds. Vascular bundle development and cell wall biosynthesis (lignin, cellulose, and hemicellulose) are coordinated by hierarchical NAC-MYB transcriptional cascades, with recent advances suggesting epigenetic regulation through DNA methylation, histone modification, and DELLA-mediated chromatin silencing in rice. We further discuss how these mechanistic insights may guide modern breeding through marker-assisted selection, genome selection, CRISPR/Cas9-mediated precision engineering, and ideotype design, providing a roadmap for developing next-generation lodging-resistant rice varieties.

## Graphical Abstract

**Figure:**
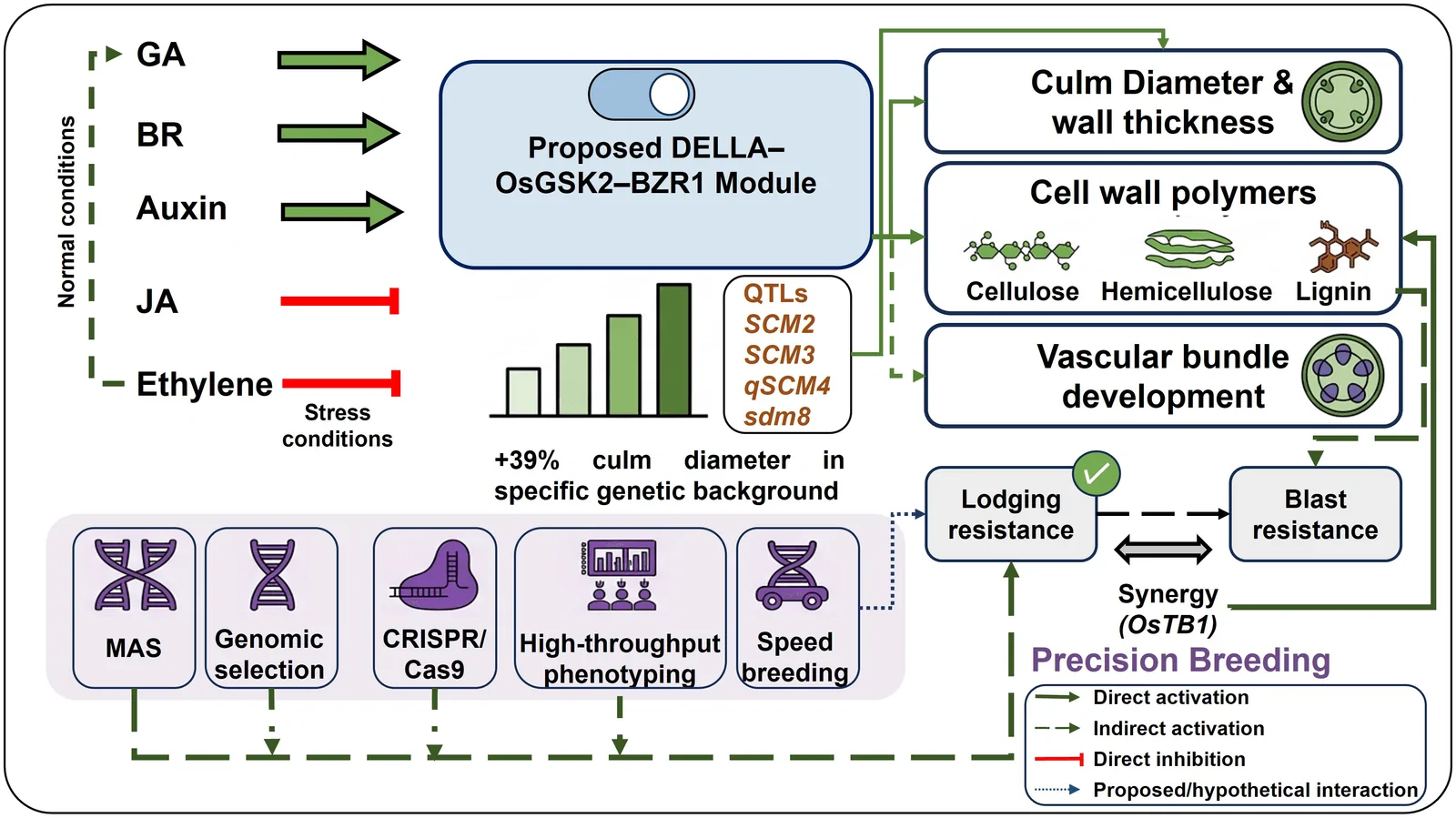
Integrative model of rice lodging resistance. Hormonal signals (GA, BR, auxin promote; JA inhibits; ethylene plays a context-dependent role: under normal conditions, it can indirectly promote GA accumulation and internode elongation via OsEATB repression; however, under stress or at high concentrations, it can also inhibit growth) are proposed to converge on the emerging DELLA–OsGSK2–BZR1 module, which regulates culm diameter, cell wall composition, and vascular bundle development. Directly demonstrated interactions are shown as solid green arrows, indirect interactions as dashed green arrows, and proposed/hypothetical interactions as dotted blue arrows. Pyramiding strong-culm QTLs (*SCM2*, *SCM3*, *qSCM4*, *sdm8*) enhances stem strength as reported in specific genetic backgrounds. Modern breeding tools (MAS, genomic selection, CRISPR/Cas9, speed breeding, high-throughput phenotyping) enable precision improvement. Lignin biosynthesis also confers synergistic resistance to blast disease via *OsTB1*.

## 1 Introduction

Rice (*Oryza sativa* L.) is a staple food for over 50% of the global population. Researchers and breeders have made extensive efforts to close the production-consumption gap, driving a 52% increase in global rice output (FAO, 2024). Grain yield is mainly determined by grain number and weight; however, achieving a high yield requires a strong culm to withstand environmental stresses, especially before harvest (Sherif et al., 2023).

Lodging is defined as the permanent displacement of a culm from its vertical posture and is considered one of the most challenging issues in cereal crops (Mullangie et al., 2024). It increases mechanical harvesting costs and can cause up to 80% yield loss under severe weather conditions, particularly when lodging occurs at the ripening stage and affects the basal internodes (Berry et al., 2004; Lang et al., 2012). Lodging also increases canopy humidity, which promotes fungal disease development (Nakajima et al., 2008). Lodging distorts carbohydrate mobilization from the culm to the sink (Setter et al., 1997; Shah et al., 2017) and damages the conducting tissues, resulting in poor grain filling (Kashiwagi and Ishimaru, 2004; Bisht et al., 2022), ultimately reducing yield.

There are three types of lodging (**Figures 1A–C**): (A) stem bending type: the culm tilts at the basal node due to heavy panicles; (B) stem breakage type: complete separation at lower internodes due to weak culm walls; and (C) root lodging type: tilting due to poor root-soil integrity (Berry et al., 2004; Islam et al., 2007; Shah et al., 2017; Bisht et al., 2022). Although root architecture affects lodging resistance (LDR) in direct-seeded rice, this review focuses on culm-related molecular mechanisms. LDR is a multifactorial trait influenced by (1) environmental factors (wind, rain, and soil), (2) morphological factors (plant height, culm length and diameter, and tiller numbers), (3) anatomical factors (culm wall thickness, vascular bundle numbers, and culm cavity), and (4) biochemical factors (cell wall chemical composition) (**Figure 1D**) (Shah et al., 2017; Bisht et al., 2022; Mullangie et al., 2024).

**Figure 1:**
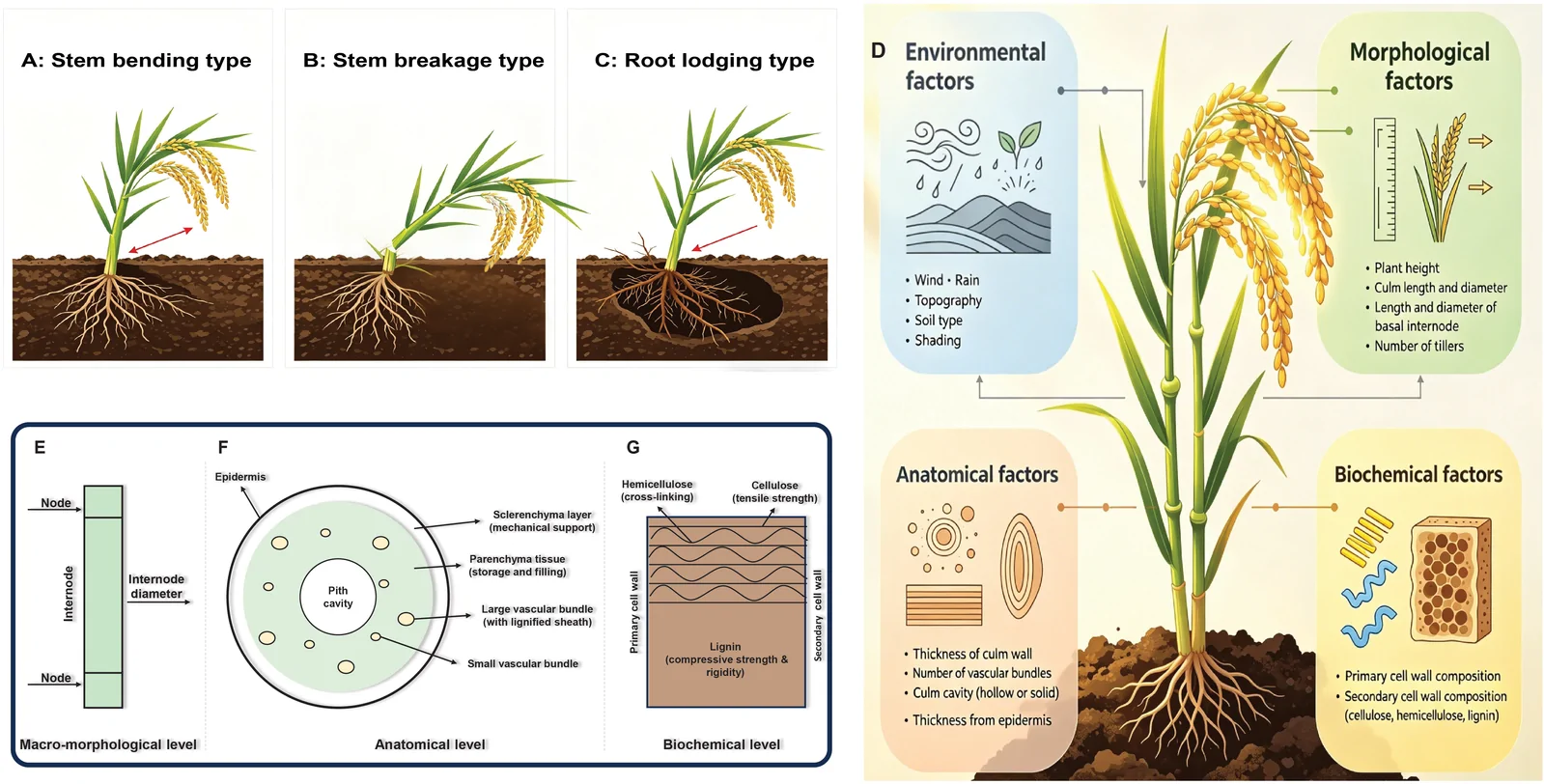
Hierarchical structural factors contributing to lodging resistance in rice culms. **(A–C)** Schematic representation of lodging type. **(D)** Overview of factors contributing to rice lodging resistance. Hierarchical structure of rice culm: from internodes to cell walls. **(E)** Key morphological features (internodes and nodes). **(F)** Anatomical tissues (epidermis, sclerenchyma, vascular bundles, pith cavity, and parenchyma). **(G)** Cell wall components (cellulose, hemicellulose, and lignin) and wall types (primary and secondary).

Breeding for LDR has evolved over three generations: reducing plant height (1960s Green Revolution), focusing on strong culms, and strengthening basal culms (Kashiwagi, 2022). This review adopts a narrative approach, integrating diverse mechanistic insights from genetic, hormonal, epigenetic, and cell wall network studies. The literature was identified through systematic searches of the PubMed, Web of Science, and Scopus databases until early 2026 using keywords including rice LDR, culm strength, cell wall biosynthesis, phytohormone regulation, QTLs, CRISPR, and epigenetics. We prioritized findings corroborated by multiple independent studies and noted the potential publication bias that is inherent in narrative reviews.

This review aims to (1) identify key genes and pathways that enhance culm strength, (2) analyze genetic, hormonal, and environmental interactions, and (3) discuss the breeding significance of these findings in rice. We offer a synthesis of molecular mechanisms for ideotype design, with an emphasis on emerging epigenetic insights and advanced technologies.

Although previous reviews have thoroughly described individual aspects of LDR, such as GA-DELLA regulation of plant height (Dai et al., 2025), brassinosteroid signaling (Clouse, 2011), cell wall composition (Liu et al., 2018), and QTL mapping (Kashiwagi et al., 2008) an integrated synthesis that connects hormonal crosstalk, epigenetic regulation, and modern breeding strategies into a single conceptual framework has been lacking. Recently published reviews (Shah et al., 2017; Mullangie et al., 2024) have summarized the factors affecting lodging but did not fully integrate the emerging DELLA-OsGSK2-BZR1 module as a central hub, nor did they incorporate the newly identified accelerator/brake transcriptional modules or the epigenetic control of cell wall genes.

A key unresolved question that underpins much of the research on culm strength is the relative contribution of lignin versus cellulose to mechanical strength. While lignin has traditionally been considered the primary determinant of compressive strength and rigidity, recent evidence suggests that cellulose microfibril orientation and hemicellulose cross-linking may be equally or more important under certain stress conditions. Resolving this debate is critical for directing future breeding and cell wall engineering efforts.

Here, we fill this gap by constructing a comprehensive, multi-scale model that spans from phytohormone signals to cell wall deposition and finally to actionable breeding schemes, with a special emphasis on how partial loss-of-function alleles (e.g., *OsTB1* in-frame mutants) can circumvent the classical yield-lodging trade-off.

## 2 Anatomical and physiological basis of culm strength

### 2.1 The hierarchical structure of the rice culm

The mechanical strength of rice culms is a critical determinant of LDR (Li et al., 2025). Its architecture demonstrates structural integrity from macro to nanoscale (**Figures 1E–G**): (1) morphological features (internode, node), (2) anatomical tissues (epidermis, sclerenchyma, vascular bundles, pith cavity), and (3) cell wall components (cellulose, hemicellulose, lignin). The internode diameter determines the culm’s physical dimensions. Furthermore, the true mechanical threshold defined by parameters such as section modulus (a geometric property reflecting resistance to bending), breaking moment (the maximum bending force a culm can withstand before failure), and bending stress (the internal stress generated within the culm tissue in response to an applied force), is governed by microscopic tissue properties (Huang et al., 2018).

The mechanical function of each structural level is directly linked to its anatomical organization. At the macro-morphological level, a larger internode diameter increases the section modulus, thereby enhancing bending resistance (Huang et al., 2018). At the tissue level, the epidermis and sclerenchyma provide structural support through their lignified cell walls (Ma et al., 2021), while vascular bundles contribute to both transport and mechanical reinforcement (He et al., 2025). The distribution and abundance of these tissues directly influence culm rigidity and breaking strength (Kashiwagi et al., 2008; Kong et al., 2013). At the cell wall level, the precise arrangement and composition of cellulose, hemicellulose, and lignin determine the tensile and compressive properties of the culm (Cosgrove, 2024). Understanding the interplay between these structural components and the physiological processes governing their synthesis is therefore essential for comprehending a plant’s ability to withstand mechanical stress (Kokubo et al., 1989; Huang et al., 2018).

### 2.2 Key anatomical features governing mechanical strength

#### 2.2.1 Culm diameter and wall thickness

The primary morphological characteristics influencing culm strength are diameter, wall thickness, and cortical fiber tissue thickness (Kashiwagi, 2022). A larger outer diameter increases the second moment of area, thereby enhancing bending resistance. Breeders select genotypes with thicker lower internodes, as they are most vulnerable to buckling (Chigira et al., 2023; Lin et al., 2025). However, thick walls must be balanced with grain productivity.

#### 2.2.2 Sclerenchyma tissue and vascular bundle architecture

Sclerenchyma cells with heavily lignified secondary walls form a rigid outer cylinder beneath the epidermis, providing the first line of defense against bending (Ma et al., 2021). Vascular bundles embedded within this cylinder serve dual transport and structural functions, each enclosed by a lignified bundle sheath. Mechanical testing has demonstrated that culm bending strength and tissue rigidity are strongly correlated with the quantity, size, and distribution of bundles, especially large bundles in the outer cortex (He et al., 2025).

#### 2.2.3 Parenchyma tissue and culm filling

Parenchyma occupies the inner cortex and pith and is involved in photosynthesis and storage (Morris et al., 2016). During maturation, the central pith degenerates, forming a hollow cavity for weight reduction; however, the degree of the remaining parenchyma (culm filling) influences the strength. Mechanical analyses have shown that lignified parenchyma contributes to compressive strength, whereas weak parenchyma creates structural weakness (Kong et al., 2013; Bisht et al., 2022).

### 2.3 Physiological drivers of culm development and maturation

Culm development follows a precise temporal program regulated by genetics, hormones, and environmental cues (Svolacchia et al., 2025). Critical events include deposition of structural carbohydrates and lignification sclerenchyma and vascular bundles walls (Kokubo et al., 1989). Plant hormones act as master regulators of these processes.

Gibberellins (GAs) are key promoters of internode elongation, primarily by stimulating cell division and expansion. However, excessive GA activity, whether through genetic mutation or exogenous application, leads to elongated, slender internodes with reduced mechanical strength, thereby increasing lodging susceptibility (Dai et al., 2025). Conversely, reduced GA levels, as achieved through the *semi-dwarf sd1* mutation, produce shorter, sturdier culms with improved LDR (Ashikari et al., 1999; Robil et al., 2024). Brassinosteroids (BRs) promote culm thickening and lignification by regulating cell wall biosynthesis genes and enhancing secondary wall deposition (Clouse, 2011; Takatsuka and Umeda, 2014; Niu et al., 2021; Chakraborty et al., 2025). BR-deficient mutants in rice exhibit shorter but thicker culms with enhanced lignification, demonstrating the critical role of BRs in balancing elongation and strength (Hong et al., 2003). Notably, some evidence for BR function in cell wall regulation has been derived from studies in *Arabidopsis* and other dicot species (Nolan et al., 2017; Cosgrove, 2024), but rice-specific studies have confirmed the conserved role of BRs in regulating culm mechanical properties (He et al., 2002; Vriet et al., 2013).

Silicon (Si) and potassium (K) are essential mineral nutrients that significantly influence culm strength. Silicon is deposited as amorphous silica phytoliths in the epidermis and sclerenchyma, forming a physical barrier that reinforces cell walls and enhances stem rigidity (Hu et al., 2022; Mullangie et al., 2024). Studies have shown that silicon application increases the breaking resistance of basal internodes and reduces lodging index by improving stem structural integrity (Mullangie et al., 2024). Potassium plays a critical role in maintaining cellular turgor, regulating stomatal movement, and facilitating carbohydrate transport from source to sink tissues (Hu et al., 2022). Adequate potassium supply enhances cellulose and lignin synthesis by promoting photosynthetic efficiency and assimilate translocation, thereby contributing to culm mechanical strength (Mullangie et al., 2024). Combined application of silicon and potassium has been shown to have synergistic effects on LDR (Hu et al., 2022).

#### 2.3.1 Agronomic and environmental factors affecting culm strength

Beyond hormonal and nutritional regulation, several agronomic and environmental factors profoundly influence culm development and LDR. Nitrogen (N) fertilization is a double-edged sword: while moderate N application promotes tillering and yield, excessive N leads to excessive vegetative growth, elongated internodes, reduced cell wall lignification, and weakened culm strength, thereby increasing lodging susceptibility (Wu et al., 2017; Zhang et al., 2026). Studies have shown that high nitrogen levels suppress lignin biosynthesis by downregulating phenylpropanoid pathway genes, resulting in reduced stem mechanical strength (Wu et al., 2017). Optimizing nitrogen application rates is therefore crucial for balancing yield and LDR.

Planting density also affects culm strength. High planting densities increase competition for light and nutrients, leading to taller, more slender plants with reduced stem diameter and wall thickness, which compromises LDR (Chigira et al., 2023; Lin et al., 2025). Conversely, appropriate planting densities promote robust culm development by reducing interplant competition and improving light interception (Lin et al., 2025). Studies have shown that increasing planting density can decrease stem-breaking strength and increase lodging index, although the extent of these effects varies among genotypes (Chigira et al., 2023).

Water management significantly influences culm development. Alternate wetting and drying irrigation, a water-saving practice, has been shown to improve LDR by promoting thicker internodes, increased stem density, and enhanced lignin accumulation (Mullangie et al., 2024). Conversely, continuous flooding or excessive soil moisture can lead to weak, elongated culms with reduced mechanical strength (Mullangie et al., 2024). Proper water management is therefore essential for optimizing culm structural integrity.

Shading (low light intensity) reduces photosynthetic activity and carbohydrate availability, leading to decreased lignin and cellulose deposition in culm cell walls (Wu et al., 2017). Shading stress also promotes stem elongation (shade avoidance syndrome) while reducing stem diameter and wall thickness, resulting in significantly increased lodging susceptibility (Zhang et al., 2026). Studies have shown that shading decreases stem-breaking strength and increases lodging index in japonica rice by reducing non-structural carbohydrate, sucrose, lignin, and cellulose accumulation in culms (Wu et al., 2017). Mitigating shading through appropriate planting density, row orientation, and canopy management is therefore critical for maintaining culm strength.

### 2.4 Synergistic effect of root traits

In direct-seeded rice systems, root-type lodging caused by insufficient anchoring strength has received increasing attention. While the primary focus of this review is on culm-related mechanisms, we briefly address root traits here to provide a more complete perspective on LDR, particularly for direct-seeded systems where root lodging is a significant concern. Evidence suggests a complementary relationship between root and culm mechanical strength; anchorage failure often occurs before stem breakage (Oladokun and Ennos, 2006). However, current research on the molecular mechanisms underlying root lodging remains relatively limited.

One known quantitative trait locus (QTL), such as *PRL4*, has been associated with LDR and is thought to indirectly affect anchoring by regulating carbohydrate accumulation at the culm base (Kashiwagi, 2022). It should be noted that the evidence linking *PRL4* directly to root anchorage is limited; its role is primarily inferred from its effects on basal culm carbohydrate content rather than from direct measurements of root anchorage strength. Further studies are needed to establish a direct causal relationship between *PRL4* and root mechanical properties.

The interaction between root architecture and culm strength remains largely hypothetical at present, although it represents a promising area for future research. Studies on root architecture genes such as *DEEPER ROOTING 1 (DRO1)* have demonstrated their importance in drought adaptation and root system architecture (Uga et al., 2013), but their direct contribution to LDR through enhanced anchorage has not been conclusively established. Future research should therefore explore potential synergies between root architecture genes and culm strength networks, with the aim of developing integrated strategies for improving LDR in direct-seeded rice systems.

## 3 Molecular regulation of culm morphological traits

The morphological architecture of rice culms is a highly organized phenotype that is regulated by a complex genetic network. Understanding this molecular regulation is critical for targeted breeding, enabling the modification of specific traits like internode length, internode diameter, and internal structure without reducing yield. These characteristics are mostly influenced by genes involved in phytohormone production, signaling, and cell fate determination, which work together in complex ways to define the physical shape of plants (**Figure 2**) (Hargrove and Cabanilla, 1979; Tanaka et al., 2023).

**Figure 2:**
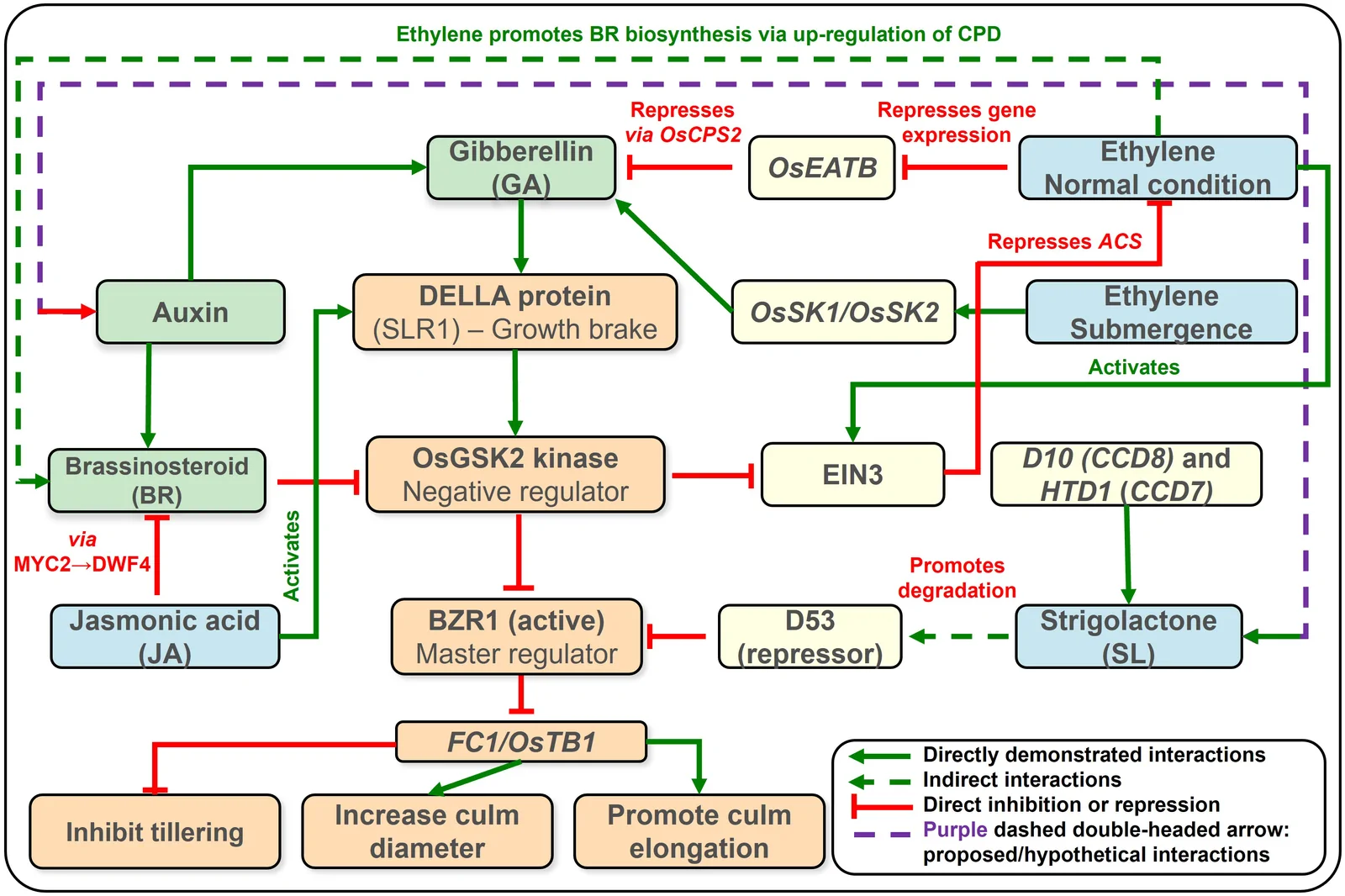
Hormonal regulation of plant height and tillering in rice. The diagram integrates the major phytohormones and their interactions that control internode elongation (plant height) and tillering. DELLA protein (SLR1) is the central growth brake. Gibberellin (GA) binds to GID1 and triggers DELLA degradation via the 26S proteasome. Brassinosteroid (BR) inactivates OsGSK2, leading to the accumulation of active BZR1, a master transcription factor for growth and cell wall synthesis. Auxin induces GA and BR biosynthesis. These three hormones synergistically promote internode elongation through the DELLA–OsGSK2–BZR1 module. Jasmonic acid stabilizes DELLA (prevents GA-induced degradation), inhibits BR biosynthesis (*via* MYC2→DWF4), and weakens BZR1 activity. Strigolactone (SL) promotes D53 degradation. D53 normally sequesters BZR1; its degradation releases BZR1, which then represses *FC1/OsTB1*. FC1/OsTB1 has three effects on rice architecture: it inhibits tillering, promotes culm elongation, and increases culm diameter. Ethylene (normal conditions) represses *OsEATB* expression. OsEATB itself represses GA biosynthesis. Thus, normal ethylene indirectly promotes GA accumulation. Ethylene (submergence) activates GA signaling via *OsSK1/OsSK2*, promoting rapid “escape” elongation. Ethylene promotes BR biosynthesis (up-regulates CPD). BR represses ethylene biosynthesis via BZR1→*ACS*. OsGSK2 acts as a negative regulator, repressing both BZR1 and EIN3. OsGSK2-mediated repression of BZR1 and EIN3 has been demonstrated in Arabidopsis and subsequently validated in rice. Thus, the figure distinguishes between pathways regulating internode elongation (plant height: GA, BR, auxin) and those regulating tillering (SL-D53-BZR1-FC1/OsTB1 axis), while also highlighting the shared DELLA–OsGSK2–BZR1 hub that integrates both processes. Arrow types: green solid arrow: directly demonstrated interactions activation/promotion; green dashed arrow: indirect interaction; red T-bar: direct inhibition/repression; purple dashed double-headed arrow: proposed/hypothetical interactions.

This review by Tanaka et al. (2023) is a key resource for readers seeking a deeper integration of the functional genomics and genetic mechanisms underlying rice plant architecture. By understanding these processes, scientists can move beyond traditional selection methods and use molecular techniques to create ideal plant types with improved culm strength and enhanced LDR.

### 3.1 Genetic control of internode length (plant height)

*Plant height is a critical factor:* Taller plants have a higher center of gravity, increasing lodging risk (Kashiwagi et al., 2008). Reducing plant height was the fastest route to improved LDR during the Green Revolution, when farmers adopted dwarf varieties carrying the *semi-dwarf 1* (*sd1*) mutation (Hargrove and Cabanilla, 1979; Khush, 1999). The *sd1* gene encodes GA20-oxidase; its mutation reduces GA levels, producing shorter, sturdier culms (Ashikari et al., 1999). Beyond these major loci, a network of QTLs fine-tunes stature by modulating meristem activity and internode elongation.

#### 3.1.1 The central role of gibberellin metabolism and signaling

The GA pathway is a central regulator of internode elongation (Wu et al., 2021). *SD1* encodes GA20-oxidase, a critical enzyme in GA biosynthesis. *sd1* alleles reducing active GA levels, were instrumental in creating high-yielding lodging-resistant varieties (Dai et al., 2025).

Downstream, the DELLA protein SLENDER RICE1 (SLR1) acts as a master repressor: in the absence of GA, SLR1 inhibits growth-promoting transcription factors; When GA binds to its receptor, SLR1 is degraded by the 26S proteosome, activating elongation genes (**Figure 2**) (Asano et al., 2009; Jung et al., 2020; Li et al., 2023). This GA-DELLA regulatory mechanism was initially characterized in Arabidopsis (Dill et al., 2001; Fu et al., 2004) and subsequently confirmed in rice (Asano et al., 2009; Li et al., 2023). The GA-DELLA regulatory node integrates with the broader DELLA-OsGSK2-BZR1 module.

#### 3.1.2 Brassinosteroid pathways as major modulators

BRs work with GAs to modulate plant architecture, with pronounced effects on cell elongation and thickening (Clouse, 2011). BR-deficient plants have shorter but thicker culms with enhanced lignification (Niu et al., 2021). GA and BR synergistically promote elongation through BRASSINAZOLE-RESISTANT1 (BZR1): BR enhances BZR1 abundance by inhibiting OsGSK2, while GA alleviates DELLA repression of BZR1 (**Figure 2**). Only simultaneous GA and BR signaling archives full BZR1 activation, optimally promoting cell elongation (He et al., 2002; Bai et al., 2012). The BZR1-mediated transcriptional network was initially characterized in Arabidopsis (He et al., 2002; Wang et al., 2002) and subsequently validated in rice (Bai et al., 2012), indicating a conserved mechanism.

#### 3.1.3 Auxin and its integration with GA/BR

In the auxin signaling pathway, the GSK3-like kinase OsGSK5 (also known as TGW3 or OsSK41) positively regulates cell elongation by relieving OsIAA10-mediated repression of OsARF4 (Ma et al., 2023). The canonical auxin pathway operates through Aux/IAA proteins (e.g., OsIAA10) that physically interact with ARF transcription factors (e.g., OsARF4) and repress their transcriptional activity. OsGSK5 directly phosphorylates OsIAA10, which has two critical consequences (Ma et al., 2023): (i) phosphorylation promotes the SCF^OsTIR1^-dependent ubiquitination and subsequent 26S proteasome-mediated degradation of OsIAA10; and (ii) phosphorylation weakens the physical interaction between OsIAA10 and OsARF4. By both depleting the OsIAA10 repressor and reducing its association with OsARF4, OsGSK5-mediated phosphorylation effectively releases OsARF4 from inhibition. The liberated OsARF4 can then activate downstream auxin-responsive genes to promote cell expansion and elongation. Thus, in this auxin sub-pathway, the kinase exerts a positive regulatory effect on auxin signaling output and cell elongation.

It is important to distinguish the roles of different GSK3/SHAGGY-like kinase family members in rice. OsGSK2 functions as a negative regulator in the BR signaling pathway by phosphorylating and inactivating OsBZR1 (He et al., 2002; Bai et al., 2012). By contrast, the distinct family member OsGSK5 (TGW3/OsSK41) functions in the auxin signaling pathway, where it exerts a positive regulatory effect as described above (Ma et al., 2023). Both pathways, BR and auxin, ultimately converge on the DELLA-OsGSK2-BZR1 module to promote elongation, but through distinct mechanisms: BR signaling inactivates OsGSK2 to relieve BZR1 repression and activate BR-responsive growth genes (He et al., 2002; Bai et al., 2012), whereas auxin signaling utilizes OsGSK5 to remove the OsIAA10 repressor and release OsARF4 for auxin-responsive gene activation (Ma et al., 2023).

It is important to distinguish between BZR1’s role in regulating tillering and internode elongation. The D53-OsBZR1 complex represses *FC1/OsTB1* expression to promote tillering (Fang et al., 2020). By contrast, BR-promoted internode elongation occurs through BZR1-mediated activation of distinct downstream targets, including cell wall biosynthesis genes (*CESA4/7/9*, *PAL, CAD, and STRONG2*). Therefore, BR signaling promotes internode elongation through BZR1-mediated activation of cell wall biosynthesis, which is consistent with its role in promoting cell elongation.

It should be noted that the link between auxin signaling and LDR is primarily indirect, as auxin regulates cell elongation and internode patterning, which in turn influence culm architecture and mechanical properties. Direct evidence for auxin-mediated LDR in rice remains limited and represents an area for future investigation.

#### 3.1.4 Strigolactones: indirect regulation via tillering

Strigolactone (SL) promotes the degradation of the D53 protein via the ubiquitin-proteasome pathway (Fang et al., 2020). D53 normally interacts with OsBZR1 to repress *FC1/OsTB1* expression, thereby promoting tillering. Upon D53 degradation, OsBZR1 loses its interacting partner, resulting in attenuated repression of the downstream gene *FC1/OsTB1*. This elevates *FC1/OsTB1* expression and consequently represses tillering (Fang et al., 2020). BR signals competitively bind to D53, thereby weakening SL inhibition (Guo et al., 2024). SL biosynthesis requires *D10* (*OsCCD8*), which is predominantly expressed in vascular tissues (Arite et al., 2007). In SL-signaling mutants, *D10* expression is elevated via feedback, whereas the *fine culm* (*fc1/Ostb1*) mutant shows no increase (Minakuchi et al., 2010), indicating that OsTB1 acts independently of this feedback loop.

In addition to its role in tillering suppression, OsTB1/FC1 also promotes culm elongation, thereby contributing to plant height regulation (Lu et al., 2013). Furthermore, the *SCM3* allele of *OsTB1* increases culm diameter, further contributing to LDR (Yano et al., 2015). Additionally, *OsTB1* interacts with OsMADS57 to repress *D14* (SL receptor), forming a miR444a-OsMADS57-OsTB1-D14 regulatory loop (Guo et al., 2013). It is important to note that the link between strigolactone signaling and LDR is indirect, primarily mediated through its effects on tillering and culm architecture (via OsTB1) rather than through direct regulation of culm mechanical properties.

Tillering and plant height are both components of plant architecture that are coordinately regulated by hormonal signals. While tillering refers to the number of lateral shoots (tillers) produced from axillary buds, plant height is determined by internode elongation. The DELLA-OsGSK2-BZR1 module integrates signals from GA, BR, and SL pathways to regulate both processes: SL signaling reduces tillering via the D53-OsBZR1-OsTB1 axis, while GA and BR signaling promote internode elongation and thus plant height (Ookawa et al., 2010b; Bai et al., 2012).

#### 3.1.5 Ethylene: a conditional regulator

Ethylene’s effect on plant height is environmentally dependent (Zhang et al., 2026). Under normal conditions, ethylene represses *OsEATB*, which in turn represses GA biosynthesis; thus, ethylene indirectly promotes GA accumulation and internode elongation (Qi et al., 2011). However, ethylene can also inhibit growth under prolonged stress or at high concentrations, reflecting its dual role as both a growth promoter and growth inhibitor depending on the physiological context (Zhang et al., 2026). Under deepwater submergence, ethylene enhances GA signaling via *OsSK1/OsSK2*, promoting rapid “escape” elongation (Dai et al., 2025). Ethylene and BR exhibit bidirectional regulation: ethylene promotes *CPD* (BR biosynthesis), while BZR1 suppresses *ACS* (ethylene biosynthesis) (Guo et al., 2024). The role of ethylene in LDR is therefore context-dependent, as it can either promote elongation (under normal conditions) or inhibit growth (under stress), indirectly influencing culm architecture and mechanical properties.

#### 3.1.6 Jasmonic acid: a stress-induced suppressor

Jasmonic acid (JA) antagonizes plant vertical growth: The JASMONATE ZIMDOMAIN PROTEIN (JAZ) proteins interact with DELLA, preventing GA-induced degradation and shortening internodes (Dai et al., 2025). Additionally, JA inhibits BR biosynthesis via MYC2 binding to the *DWF4* promoter and weakens BR activity (Zhang et al., 2023). During pathogen infection, JA accumulation diverts resources from growth to defense.

#### 3.1.7 Summary of hormonal network

Rice plant height is determined by the balance between growth-promoting hormones (GA, BR, and auxins) and growth-inhibiting hormones (JA and, under certain conditions, ethylene and SL). Critically, ethylene is not simply a growth inhibitor; it is a conditional regulator. Under normal conditions, ethylene can indirectly promote GA accumulation and internode elongation by repressing *OsEATB* (Qi et al., 2011); however, under prolonged stress or at high concentrations, ethylene can also inhibit growth (Zhang et al., 2026). This context-dependent role resolves the apparent contradiction: ethylene promotes elongation under normal conditions but inhibits growth under stress. SL mainly affects plant height indirectly by regulating tillering through the D53-OsBZR1-OsTB1 axis. These signals converge at DELLA and BZR1 nodes, providing breeders with molecular tools for precise height modification (**Figure 2**).

### 3.2 Genetic control of culm diameter and internode structure

The overall mechanical performance of a rice culm is strongly influenced by its cross-sectional geometry, which can be classified into three principal types: filled (solid, no cavity), hollow with a thick wall, and hollow with a thin wall (**Figure 3A**).

**Figure 3:**
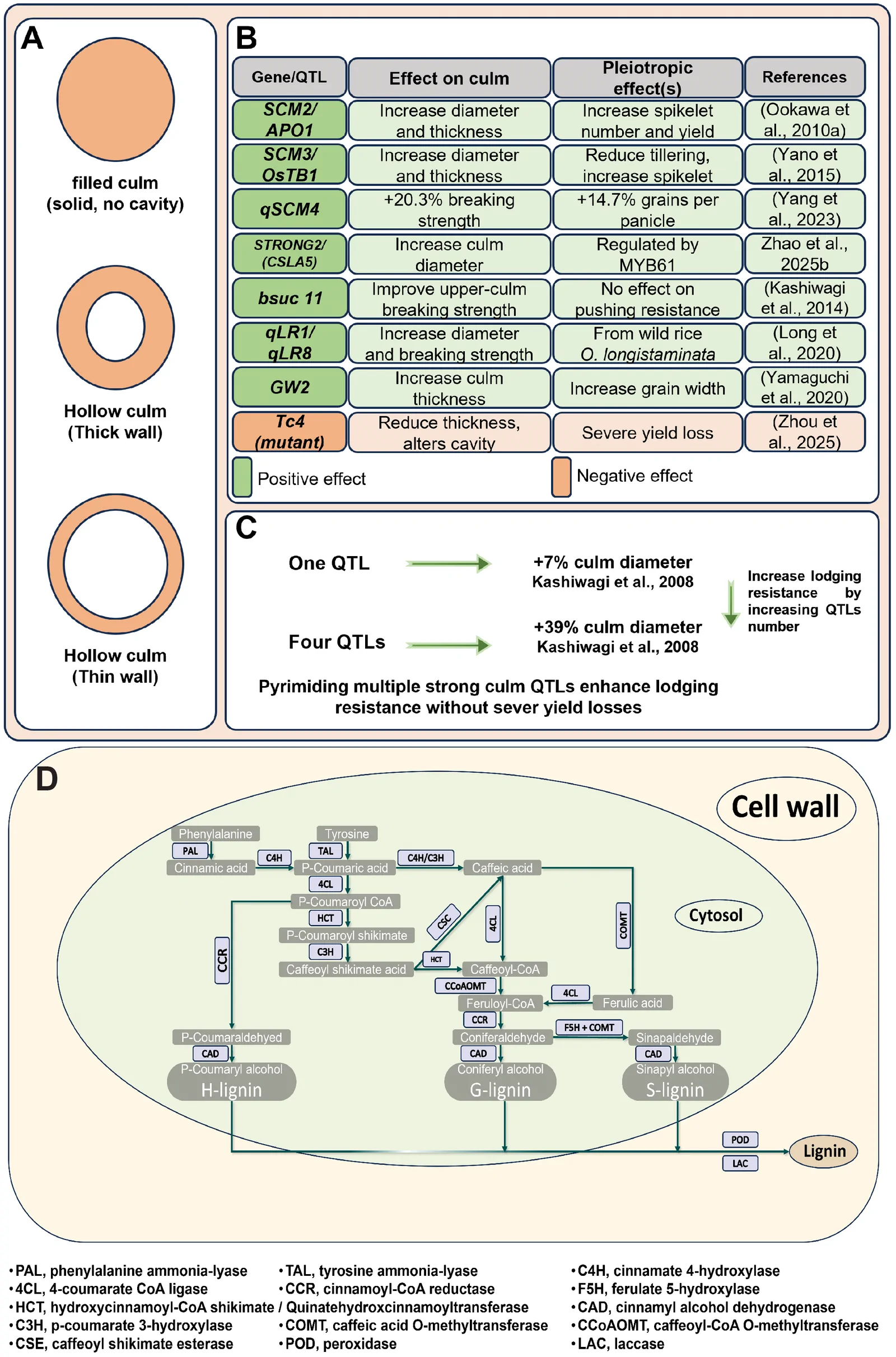
Genetic control of culm morphology, QTL pyramiding, and lignin biosynthesis in rice. **(A)** Three principal cross-sectional- types of rice culms: filled culm (solid, no cavity), hollow culm with a thick wall, and hollow culm with a thin wall and large diameter. This morphological diversity provides targets for genetic improvement. **(B)** Major QTLs and genes controlling culm diameter and mechanical strength. The table lists each gene/QTL, its effect on culm properties, pleiotropic effects on agronomic traits, and key references. Green rows indicate beneficial alleles; the orange row (*tc4*) represents a detrimental mutant. **(C)** Pyramiding of multiple strong-culm QTLs has an additive effect on culm diameter - combining four QTLs increased diameter by 39% compared to 7% for a single QTL in specific genetic background, demonstrating that stacking favorable alleles enhances LDR without a severe yield penalty. **(D)** General biosynthesis pathway of lignin in higher plants.

#### 3.2.1 Major QTLs and genes

Culm diameter is a primary target for LDR breeding (Kashiwagi et al., 2008). A rich genetic architecture underlies variation in culm thickness and strength. Major QTLs with substantial effects have been identified (**Table 1**; **Figure 3B**). Among the cloned genes with functional validation, *STRONG CULM 2 (SCM2)*, an allele of *ABERRANT PANICLE ORGANIZATION1 (APO1)*, which increases culm diameter while positively affecting panicle organization and yield; its function has been validated through transgenic complementation (Ookawa et al., 2010a). Similarly, *SCM3* (an allele of *RICE TEOSINTE BRANCH1/OsTB1)* increases diameter and modulates tillering, with validation through CRISPR/Cas9-mediated promoter editing (Yano et al., 2015; Cui et al., 2020). *OsTB1* is directly controlled by IPA1 via binding to a GTAC motif in its promoter, linking the miR156-IPA1 module to shoot branching and culm development (Lu et al., 2013). As discussed in Section 3.1.4, OsTB1/FC1 also promotes culm elongation, thereby contributing to plant height regulation, in addition to its role in tillering suppression and culm diameter increase.

**Table 1:** Key genes regulating vascular bundle development, parenchyma cell proliferation, and internal stem structure in rice.

Gene	Protein family/function	Effect on culm and vascular traits	Effect on lodging resistance	Evidence type	Reference(s)
*DEP1 (qLVB9)*	G protein γ subunit	Increases number of both large and small vascular bundles in the panicle neck; enhances grain yield	Positive (improves stem mechanical support via more vascular bundles)	Anatomical + Mechanical	(Fei et al., 2019; Wang et al., 2024)
*APO1/SCM2 (HII allele)*	F-box protein; promoter variant	Increases large vascular bundle number by ~15% and peduncle diameter by ~9%; improves harvest index	Positive (thicker peduncle and more bundles increase pushing resistance)	Direct lodging + Mechanical	(Ookawa et al., 2010a; Terao et al., 2010)
*NAL1/SPIKE*	Serine protease	Affects vein patterning and polar auxin transport; regulates vascular bundle area and phloem area	Positive (enhanced vascular area improves assimilate transport and stem strength)	Anatomical + Mechanical	(Qi et al., 2008; Fujita et al., 2013; Zhai et al., 2018)
*AVB/NRL3*	a land plant conserved protein with unknown functions	Promotes procambium establishment; knockout reduces large vascular bundles by ~61% and small bundles by ~60% in internodes	Positive (increased vascular bundle number correlates with stronger culm)	Mechanical + Anatomical	(Ma et al., 2017; Chen et al., 2019)
*OsCOMT*	Caffeic acid O-methyltransferase (melatonin biosynthesis)	Increases vascular bundle number and area via melatonin-cytokinin axis; overexpression enhances, knockout reduces	Positive (more and larger vascular bundles directly improve mechanical strength)	Mechanical + Anatomical	(Huangfu et al., 2022)
*TAL*	Transaldolase (horizontally transferred from bacteria)	Expressed in phloem; knockdown reduces outer vascular bundle number and adjacent parenchyma cell layers	Positive (proper vascular patterning required for normal culm rigidity)	Anatomical + Inferred	(Yang et al., 2015)
*DVB1*	Mic10 family (mitochondrial cristae organization)	Maintains mitochondrial structure; mutant reduces large vascular bundles by ~69% and small by ~70% in culm	Positive (mitochondrial function indirectly supports vascular development and stem integrity)	Anatomical + Inferred	(Zhang et al., 2021)
*OsYABBY1/2*	YABBY transcription factor	Promotes differentiation of mestome sheath and abaxial sclerenchyma (thick-walled mechanical tissues)	Positive (directly increases lignified mechanical support around bundles and in outer stem)	Anatomical + Mechanical	(Toriba et al., 2007)
*OsYABBY4*	YABBY transcription factor	Overexpression reduces large vascular bundle number while increasing tiller number (trade-off)	Negative (fewer vascular bundles weaken culm; trade-off with tillering)	Anatomical + Inferred	(He et al., 2025)
*RF2a*	bZIP transcription factor	Essential for normal vascular bundle organization and phloem development; antisense causes disordered bundles	Positive (proper bundle patterning is critical for stem load-bearing capacity)	Anatomical + Mechanical	(Yin et al., 1997)
*OsOFP2*	Ovate family protein	Interacts with KNAT7/BLH6; alters radial position of vascular bundles and affects lignin and GA biosynthesis	To be determined (inward displaced bundles may reduce support; further studies needed)	Anatomical + Inferred	(Schmitz et al., 2015)
*OsEXO70A1 (RLS2)*	Exocyst subunit (vesicle trafficking)	Required for vascular bundle integrity and secondary cell wall deposition; mutants have irregular xylem, reduced cellulose/lignin	Positive (vesicle trafficking is essential for normal cell wall strength and bundle structure)	Mechanical + Anatomical	(Tu et al., 2015)
*BC12*	Kinesin-4	Modulates cell cycle progression and secondary wall properties; affects stem morphology and brittleness	Positive (proper cell cycle and wall properties prevent brittle stems)	Mechanical + Anatomical	(Zhang et al., 2010)
*SSC/RFL/APO2*	Putative uncharacterized protein	Controls solid culm (filled pith) vs. hollow culm; reduces medullary cavity formation	Positive (solid culm resists bending and breaking better than hollow)	Direct lodging + Mechanical	(Wang et al., 2017)

#### 3.2.2 Mapped QTLs with significant effects

Several QTLs have been mapped in biparental populations and validated across multiple environments. For example, *qSCM4* was identified in a recombinant inbred line (RIL) population derived from a cross between a strong-culm landrace and an elite cultivar; under field conditions, this QTL boosts LDR by 20.3% and grain number by 14.7% (Yang et al., 2023). Other mapped QTLs include *sdm8*, and *qCD7-2*, which have been consistently detected in multiple mapping populations and environments (Kashiwagi et al., 2008; Chigira et al., 2023).

#### 3.2.3 Pyramiding of strong culm QTLs

Combining four stem diameter loci (*sdm1, sdm7, sdm8*, and *sdm12*) in a mapping population derived from a cross between Koshihikari (a japonica variety with thin culms) and a strong-culm landrace increased culm diameter by up to 39% and enhanced pushing resistance under field conditions (Kashiwagi et al., 2008), demonstrating that stacking favorable alleles improves LDR without severe yield penalties (**Figure 3C**). In contrast to these beneficial alleles, the *tc4 (thin culm 4)* mutant represents a detrimental allele with a negative effect on LDR. The *tc4* mutant, which is allelic to *RFL/APO2/SSC*, exhibits thinner culms with thicker inner walls and a smaller lumen diameter, resulting in a reduced section modulus and significantly weakened breaking strength (Zhou et al., 2025). Unlike typical brittle mutants that show reduced cellulose content, *tc4* displays increased cellulose and hemicellulose levels, suggesting that its weakened mechanical strength is primarily due to morphological changes. Specifically, reduced culm diameter and altered wall thickness rather than altered cell wall composition. This negative-effect mutant highlights the critical importance of culm morphology and structural integrity for LDR.

GWAS associations and candidate genes: Genome-wide association studies have identified candidate genes associated with culm diameter. For example, *STRONG2 (CSLA5)*, a mannan synthase gene regulated by MYB61, was identified through GWAS and subsequently validated by overexpression and knockout analyses in rice, confirming its role in increasing culm diameter (Zhao et al., 2025b). Pleiotropic genes such as *OsPRR37* and *GW2* have been associated with both culm strength and agronomic traits through GWAS, although their functional validation in the context of LDR requires further investigation traits (Yamaguchi et al., 2020; Guo et al., 2021).

The QTL *bsuc11 (breaking strength of upper culms on chromosome 11)* simultaneously increases the breaking strengths of the top three internodes without affecting internode length, and functions by preventing the natural decline in culm strength during grain filling through increased holocellulose (mainly α-cellulose) content (Kashiwagi, 2014; Kashiwagi et al., 2016). A near-isogenic line carrying *BSUC11a*, the main effector sub-QTL, showed improved upper-culm strength and greater lodging resistance without compromising plant length or yield under field conditions (Kashiwagi and Munakata, 2025).

#### 3.2.4 Novel sources and validation status

Wild rice (*O. longistaminata*) provides novel QTLs (*qLR1* and *qLR8*) that have been mapped but await functional validation and cloning (Long et al., 2020). The validation status of each gene/QTL is summarized in **Figure 3B** and **Table 1**, with cloned and functionally validated genes distinguished from mapped QTLs and GWAS associations requiring further validation.

### 3.3 Genes regulating vascular bundle development, parenchyma cell proliferation, and internal structure of the stem

#### 3.3.1 Genes controlling vascular bundle number

Vascular bundle development is controlled by genes that regulate procambial cell initiation and differentiation (Jung et al., 2008). Key genes include *DENSE AND ERECT PANICLE 1* (*DEP1*) (also known as *qLVB9*), which increases large and small vascular bundle numbers in the neck node; this anatomical enhancement is associated with improved mechanical properties (Fei et al., 2019; Wang et al., 2024). *APO1/SCM2* promotes cell proliferation, increasing bundle quantity, stem diameter, and pith filling; mechanical testing has confirmed that these anatomical changes contribute to increased pushing resistance (Ookawa et al., 2010a). The *NARROW LEAF 1* (*NAL1*) gene affects vein patterning and auxin polar transport (Qi et al., 2008; Fujita et al., 2013). The *ABNORMAL VASCULAR BUNDLES* (*AVB*) gene, encodes a conserved protein downstream of auxin signaling that promotes procambium establishment (Ma et al., 2017). Other notable genes include *OsCOMT* (melatonin-cytokinin axis promoting bundle proliferation) (Huangfu et al., 2022), *TRANSALDOLASE-LIKE* (*TAL*) (horizontally transferred transaldolase essential for vascular patterning) (Yang et al., 2015), and *Decreased Vascular Bundle 1* (*DVB1*) (mitochondrial MICOS component linking mitochondrial structure to vascular development) (Zhang et al., 2021). Further studies have identified QTLs controlling vascular bundle quantity, such as *qLVN6* (candidate gene *OsARF18*), *qSVN7* (candidate gene *Ghd7.1*), and *qSVA8.1* (candidate gene *OsFIE2*) (Zhai et al., 2018).

#### 3.3.2 Transcriptional regulators of vascular differentiation

##### 3.3.2.1 Transcriptional regulators

OsYABBY1 promotes differentiation of thick-walled mechanical tissues around bundles (Toriba et al., 2007), while OsYABBY4 overexpression reduced bundle number but increased tiller number, revealing a trade-off (He et al., 2025). RF2a is a basic leucine zipper (bZIP) transcription factor essential for normal vascular bundle differentiation (Yin et al., 1997), and rice *Ovate Family Protein 2* (*OsOFP2*) interacts with KNOX proteins to alter bundle positioning and affect lignin biosynthesis (Schmitz et al., 2015).

##### 3.3.2.2 Solid vs. hollow culms

*SCM2/APO1* enhances stem solidity by reducing pith cavity through cell proliferation (Ookawa et al., 2010a). *SSC* and *BC12* also influence the balance between elongation and cavity formation (Zhang et al., 2010; Wang et al., 2017). **Table 1** presents a comprehensive list of these genes.

## 4 Molecular regulation of culm chemical composition and microstructure

### 4.1 Biosynthesis and deposition of cell wall polymers

The cell wall is a composite of cellulose microfibrils embedded in a matrix of hemicellulose and lignin (Bisht et al., 2022; Guo et al., 2024).

#### 4.1.1 Lignin biosynthesis

Lignin provides compressive strength, rigidity, and hydrophobicity, particularly in sclerenchyma and vascular bundles, and is a critical determinant of culm mechanical strength and LDR (Liu et al., 2018; Mili et al., 2022). The pathway proceeds from phenylalanine through PAL, CCR, and CAD enzymes to produce H, G, and S lignin monomers (**Figure 3D**; **Table 2**) (Yoon et al., 2015). Rice has 90 lignin biosynthesis genes belonging to ten gene families (Shad et al., 2024). Mutations in genes encoding key lignin biosynthetic enzymes reduce lignin content and cause lodging-susceptible phenotypes. For example, mutations in *FLEXIBLE CULM 1* (*FC1*; encoding cinnamyl alcohol dehydrogenase, CAD), not to be confused with *FC1/OsTB1* (*FINE CULM 1*) a tillering regulator discussed in Section 3.1.4, or in *CCR* genes reduce lignin content, leading to weakened culm mechanical strength and increased lodging susceptibility in rice (Li et al., 2009). It should be noted that while CCR downregulation has been extensively studied in poplar (Leplé et al., 2007), direct evidence in rice is more limited; however, the available rice data support a conserved role for *CCR* in lignin biosynthesis and culm mechanical strength.

**Table 2:** Key genes involved in cell wall biosynthesis, transcriptional regulation, and hormone signaling affecting culm mechanical strength in rice.

Gene	Encoded protein/family	Role in cell wall/culm trait	Effect on lodging resistance	Reference(s)
Cellulose synthase *(CESA)* genes				
*OsCESA1*	Cellulose synthase (primary wall)	Primary wall cellulose synthesis; downregulated by shading	Positive (reduction decreases strength)	(Wu et al., 2017)
*OsCESA3*	Cellulose synthase (primary wall)	Primary wall cellulose synthesis; downregulated by shading	Positive (reduction decreases strength)	(Wu et al., 2017)
*OsCESA8*	Cellulose synthase (primary wall)	Primary wall cellulose synthesis; downregulated by shading	Positive (reduction decreases strength)	(Wu et al., 2017)
*OsCESA4*	Cellulose synthase (secondary wall)	Secondary wall cellulose synthesis; upregulated by OsMYB103L	Positive (based on loss-of-function mutant phenotype)	(Tanaka et al., 2003; Yang et al., 2014; Wu et al., 2017)
*OsCESA7*	Cellulose synthase (secondary wall)	Secondary wall cellulose synthesis; upregulated by OsMYB103L	Positive (based on loss-of-function mutant phenotype)	(Tanaka et al., 2003; Yang et al., 2014; Wu et al., 2017)
*OsCESA9*	Cellulose synthase (secondary wall)	Secondary wall cellulose synthesis; mutation reduces strength	Positive (based on loss-of-function mutant phenotype)	(Tanaka et al., 2003; Yang et al., 2014; Wu et al., 2017)
*BC19 (CESA4 allele)*	Cellulose synthase catalytic subunit	Secondary wall cellulose synthesis (dominant brittle mutant)	Positive (dominant brittle mutant phenotype)	(Ma et al., 2021)
*BC1*	COBRA-like protein	Cellulose assembly	Positive (based on loss-of-function mutant phenotype)	(Li et al., 2003)
Hemicellulose and lignin biosynthesis genes				
*FLEXIBLE CULM 1 (FC1)*	Cinnamyl alcohol dehydrogenase (CAD)	Lignin biosynthesis (final step)	Positive (based on loss-of-function mutant phenotype)	(Li et al., 2009)
*OsCAD2*	Cinnamyl alcohol dehydrogenase	Lignin biosynthesis; downregulated by shading	Positive	(Wu et al., 2017)
*OsPAL*	Phenylalanine ammonia-lyase	Lignin biosynthesis (first committed step); downregulated by shading	Positive	(Wu et al., 2017)
*OsCOMT*	Caffeic acid O-methyltransferase	Lignin biosynthesis (S-lignin pathway); downregulated by shading	Positive	(Wu et al., 2017)
*OsCCOAOMT*	Caffeoyl-CoA O-methyltransferase	Lignin biosynthesis; downregulated by shading	Positive	(Wu et al., 2017)
*OsCCR*	Cinnamoyl-CoA reductase	Lignin biosynthesis (rate-limiting step); downregulated by shading	Positive	(Wu et al., 2017)
*BC3*	Dynamin OsDRP2B	Secondary cell wall synthesis (vesicle trafficking)	Positive	(Hirano et al., 2010)
*CSLF6*	Cellulose synthase-like F6	Mixed-linkage glucan (hemicellulose) synthesis	Positive	(Vega-Sánchez et al., 2012)
*STRONG2 (CSLA5)*	Mannan synthase (CSLA)	Mannan and cellulose content; sclerenchyma thickness	Positive (overexpression enhances strength)	(Zhao et al., 2025b)
Hormone-related and vesicle trafficking genes				
*D2/CYP90D2*	Cytochrome P450 (BR biosynthesis)	C-3 oxidation in BR synthesis; mutant has reduced BR, altered cell wall	Positive (BR promotes wall thickening; mutant reduces strength)	(Hong et al., 2003)
*OsGA20ox2 (SD1)*	Gibberellin 20-oxidase	GA biosynthesis; semi-dwarf allele reduces GA; high GA suppresses secondary wall genes	Negative (reduced GA increases wall thickness)	(Sasaki et al., 2002; Robil et al., 2024)
*OsEXO70A1*	Exocyst subunit (vesicle trafficking)	Required for vascular bundle integrity and secondary cell wall deposition; mutants have irregular xylem and reduced cellulose/lignin	Positive	(Tu et al., 2015)
*STRONG1 (MAP70)*	Microtubule-associated protein 70	Microtubule organization; cellulose deposition	Negative (knockout increases strength)	(Zhao et al., 2025a)
Transcription factors and regulators				
*OsMYB61*	R2R3-MYB transcription factor	Activates STRONG2 and other wall genes	Positive (overexpression enhances; mutants reduce strength)	(Chen et al., 2023; Zhao et al., 2025b)
*OsMYB103L*	R2R3-MYB transcription factor	Activates OsCESA4,7,9 and lignin genes	Positive (based on knockdown/overexpression studies)	(Yang et al., 2014)
*OsNAC31*	NAC transcription factor	Primary regulator of secondary wall genes	Positive	(Chen et al., 2023)
*OsbHLH002*	bHLH transcription factor	Partner of OSH1; activates OsMYB61	Positive	(Chen et al., 2023)
*OSH1*	Class I KNOX homeodomain protein	Cooperates with OsbHLH002 to activate OsMYB61	Positive	(Chen et al., 2023)
*OsKNAT7*	KNOX transcription factor	Repressor of OsMYB61 expression	Negative	(Chen et al., 2023)
*SLR1*	DELLA protein	GA signaling repressor; inhibits OsbHLH002-OSH1	Negative (loss-of-function reduces height; gain-of-function increases strength)	(Chen et al., 2023)

#### 4.1.2 Cellulose synthesis

Cellulose microfibrils are synthesized by cellulose synthase complexes (CSCs), which are rosette-shaped hexamers of 18 CESA subunits that move along the plasma membrane, guided by cortical microtubules (**Figure 4A**) (Somerville, 2006; Pedersen et al., 2023). Rice secondary wall-specific *OsCESA4*, *OsCESA7*, and *OsCESA9* are required for normal culm cellulose synthesis; loss-of-function mutants reduce cellulose to 8.9% of wild type level (Tanaka et al., 2003). Other brittle culm genes (*BC1*, *BC3*) encode proteins essential for cellulose assembly and microfibril packaging order and are major contributors to tensile strength (Salem et al., 2023).

**Figure 4:**
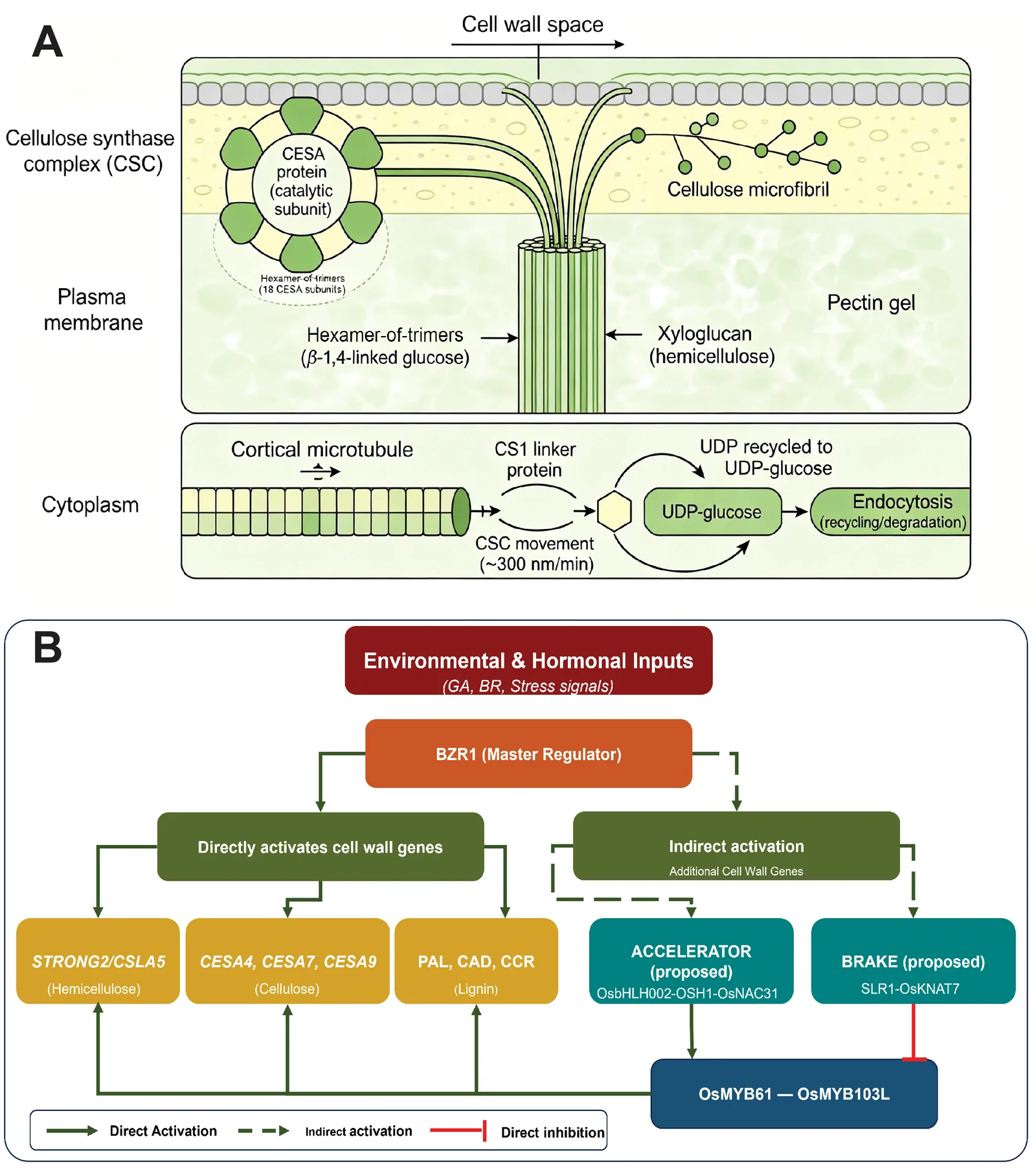
Cellulose synthesis and hierarchical transcriptional regulation in rice. **(A)** Model of cellulose synthesis at the plasma membrane. The cellulose synthase complex (CSC) is a hexamer of trimers composed of 18 CESA protein subunits embedded in the plasma membrane. UDP-glucose serves as the substrate for cellulose synthesis in the cytoplasm. The CSC moves along the plasma membrane at approximately 300 nm/min, extruding β-1,4-linked glucan chains into the cell wall space, where they assemble into cellulose microfibrils. Cortical microtubules guide CSC movement via the linker protein cellulose synthase interactive protein 1 (CSI1). Xyloglucan (a hemicellulose) cross-links cellulose microfibrils within the pectin gel matrix. The CSC is recycled or degraded via endocytosis. UDP, uridine diphosphate. **(B)** Hierarchical regulatory network of secondary cell wall biosynthesis in rice. Environmental and hormonal inputs (GA, BR, stress signals such as shading and nutrient availability) converge on BZR1, a master regulator that directly activates secondary wall biosynthesis genes including *STRONG2/CSLA5* (hemicellulose), *CESA4/7/9* (cellulose), and *PAL, CAD, CCR* (lignin) via direct activation (green solid arrows). Additional cell wall genes are indirectly regulated through downstream transcriptional cascades (green dashed arrows). Two counteracting modules, the accelerator (proposed) (OsbHLH002-OSH1-OsNAC31) and the brake (proposed) (SLR1-OsKNAT7), modulate OsMYB61 expression. MYB master regulators (OsMYB61 and OsMYB103L) integrate signals from the accelerator/brake modules and regulate downstream target genes. Direct inhibition is indicated by red T-bars. Thicker arrows denote stronger regulation. Arrow types: green solid arrow = direct activation; green dashed arrow = indirect activation; red T-bar = direct inhibition.

#### 4.1.3 Hemicellulose

Hemicellulose cross-link cellulose microfibrils to the lignin matrix (Rao et al., 2023). In rice, they are composed of xyloglucan, xylan, mannan, and mixed linkage glucans polymerization (Wen et al., 2025). Critically, a mannan synthase directly targeted by MYB61 increases mannose and cellulose level, enhancing sclerenchyma development and culm diameter (Zhao et al., 2025b).

### 4.2 Genetic and transcriptional networks steering wall composition

The synthesis of cell wall components is not a passive process but is tightly controlled at the transcription level. A complex network of transcription factors and hormonal signals coordinates the expression of biosynthetic genes to ensure proper wall formation during development and in response to environmental cues (Zhang et al., 2021; Gu and Rasmussen, 2022; Cosgrove, 2024).

#### 4.2.1 Transcription factors

Secondary wall biosynthesis is orchestrated by the NAC-MYB cascade (**Figure 4B**) (Nakano et al., 2015). *OsNAC31* acts as a primary regulator that activates downstream biosynthetic genes (Chen et al., 2023). OsMYB61 directly activates *STRONG2* (*CSLA5*) to control culm diameter and LDR (Zhao et al., 2025b). OsMYB103L binds to the promoters of lignin (*PAL*, *CAD*, *CCR*) and cellulose (*CESA*) genes (Yang et al., 2014; Wu et al., 2024).

Recent evidence has identified additional regulators that form a proposed “accelerator/brake” module controlling *OsMYB61* expression (Chen et al., 2023). The accelerator module (OsbHLH002-OSH1-OsNAC31) promotes secondary wall formation, whereas the brake module (SLR1/DELLA-OsKNAT7) represses it. It should be noted that this model is based on relatively limited recent evidence (Chen et al., 2023) and should therefore be considered an emerging framework requiring further validation in diverse genetic backgrounds and environments. STRONG1 (OsMAP70) influences cellulose deposition via microtubule orientation (Zhao et al., 2025a).

#### 4.2.2 Hormonal interplay in wall remodeling

BR directly influences NAC and MYB transcription factors (Nolan et al., 2017; Chakraborty et al., 2025), driving cellulose and lignin gene expression. High GA levels suppress secondary wall synthesis genes via DELLA interaction with the OsbHLH002-OSH1 module (Chen et al., 2023; Robil et al., 2024). Stress, such as shading, downregulates hormone signaling, thereby reducing wall biosynthesis gene expression (Wu et al., 2017). Hormonal crosstalk ensures that cell wall deposition is integrated into overall growth and stress responses. **Table 2** presents a comprehensive list of these genes.

## 5 Integrative genetic pathways and key regulators of culm strength

### 5.1 Brassinosteroid signaling as a core integrator of morphology and composition

BR signaling is a central hub integrating LDR traits through dual regulation of culm morphology and cell wall composition (Clouse, 2011). Activation begins with OsBRI1, followed by OsBAK1 and OsGSK2, ultimately modulating BZR1 activity (Vriet et al., 2013). BZR1 activates downstream genes through direct and indirect mechanisms. Direct BZR1 targets (genes to which BZR1 directly binds and activates) include secondary wall biosynthesis genes such as *CESA4/7/9* (cellulose synthase), *PAL*, *CAD* (lignin biosynthesis), and *STRONG2* (mannan synthesis) (**Figure 4B**). Indirectly regulated genes (those affected through downstream transcriptional cascades or secondary effects) include additional cell wall-related genes whose expression is modulated by BZR1-regulated transcription factors or signaling intermediates (Yang et al., 2014; Zhao et al., 2025b). BR-defective mutant *d2* exhibits altered morphology and mechanical strength, confirming the physiological significance of BR signaling in culm development (Hong et al., 2003).

Despite significant progress, several mechanistic debates remain unresolved. One contentious area concerns the relative contribution of lignin versus cellulose to culm mechanical strength. While early studies emphasized lignin as the primary determinant (Liu et al., 2018; Shad et al., 2024), recent evidence suggests that cellulose microfibril orientation and hemicellulose cross-linking may be equally or more important under certain stress conditions (Nakano et al., 2015; Zhao et al., 2025b). This debate has been dissected using multiple complementary approaches, including mutant analysis, advanced imaging, and biochemical correlation studies.

#### 5.1.1 Mutant-based approaches

Cellulose-deficient mutants (e.g., *cesa4, cesa7, cesa9, bc1, bc3*) and lignin-deficient mutants (e.g., *fc1/cad2, ccr*) have been used to compare mechanical phenotypes. Studies show that cellulose deficiency generally reduces tensile strength, while lignin deficiency primarily affects compressive strength and rigidity, suggesting distinct but complementary roles for each polymer (Tanaka et al., 2003; Li et al., 2009; Wu et al., 2017).

#### 5.1.2 Advanced imaging techniques

Confocal Raman microscopy enables *in situ* visualization of lignin, cellulose, and hemicellulose distribution at high resolution, allowing correlation of spatial distribution with mechanical properties (Huang et al., 2025). X-ray micro-CT provides three-dimensional structural information on cell wall architecture and vascular bundle arrangement (Wu et al., 2019). These techniques have revealed that the spatial organization of cell wall polymers may be as important as their total abundance.

#### 5.1.3 Chemical and enzymatic treatments

Sequential extraction and enzymatic digestion methods have been used to isolate the contributions of individual wall components, demonstrating that the removal of lignin reduces compressive strength, while cellulose removal primarily affects tensile strength (Kokubo et al., 1989; Bisht et al., 2022).

#### 5.1.4 Biochemical correlation studies

Correlation analyses between lignin/cellulose content and mechanical parameters (bending stress, breaking force, section modulus) across diverse rice varieties have shown that both polymers contribute to strength, with their relative importance varying by tissue type and developmental stage (Liu et al., 2018).

A second area of debate centers on whether the DELLA-OsGSK2-BZR1 module acts primarily through transcriptional regulation of cell wall genes or through additional post-translational mechanisms. The transcriptional model is supported by direct evidence that BZR1 binds to the promoters of cell wall biosynthetic genes, including *CESA4/7/9, PAL, CAD*, and *STRONG2*, and activates their expression (He et al., 2002; Bai et al., 2012; Zhao et al., 2025b). This model is well-established in both Arabidopsis and rice. However, it does not fully explain the rapidity of some BR-induced mechanical changes or the observed effects on cell wall architecture that occur independently of transcriptional changes. The post-translational/structural model proposes that BR signaling may also influence wall properties through microtubule orientation and cellulose synthase complex dynamics, potentially via direct phosphorylation of cellulose synthase components or microtubule-associated proteins (Nakano et al., 2015; Zhao et al., 2025a). This model is supported by studies showing that BR signaling affects cellulose microfibril orientation and that STRONG1 (OsMAP70) influences cellulose deposition via microtubule orientation (Zhao et al., 2025a). However, direct evidence linking BR signaling to these post-translational effects in rice remains limited, and the relative contribution of transcriptional versus post-translational mechanisms has not been systematically quantified. The most parsimonious interpretation is that both mechanisms operate in parallel, with transcriptional regulation providing sustained changes in cell wall composition and post-translational mechanisms enabling rapid adaptive responses. These competing models highlight the need for integrated approaches combining genetic, biochemical, and imaging techniques to fully resolve the mechanistic basis of culm strength.

### 5.2 The strong culm ideotype and its regulatory logic

The strong-culm ideotype integrates multiple trait components (culm diameter, wall thickness, vascular bundle number, and cell wall composition) into a unified architectural framework. Rather than targeting individual traits in isolation, the ideotype approach seeks to coordinately enhance multiple mechanical properties through the selection or engineering of pleiotropic regulatory hubs.

A key feature of the strong-culm ideotype is the identification of hub genes that simultaneously regulate culm strength and yield, thereby overcoming the classical trade-off between these traits. For example, *SCM2/APO1* increases both culm diameter and spikelet number (Ookawa et al., 2010a). Similarly, *SCM3/OsTB1* integrates culm diameter regulation with tillering control, and its *SCM3* allele represents a natural variant that enhances LDR without compromising yield (Yano et al., 2015; Cui et al., 2020). The existence of such hub genes suggests that the strong-culm ideotype is not merely a collection of independent traits but rather an emergent property of a coordinated regulatory network.

The regulatory logic underlying the ideotype is increasingly understood through the lens of the DELLA-OsGSK2-BZR1 module, which integrates hormonal signals to coordinately regulate culm elongation, thickening, and cell wall deposition (**Figure 2**). QTL pyramiding, stacking favorable alleles at multiple loci, represents a practical strategy for engineering the ideotype, with additive effects on culm diameter and LDR (Kashiwagi et al., 2008; Yang et al., 2023). Additional loci such as *DP1* further contribute to the ideotype by modulating cell wall composition, increasing both hemicellulose and cellulose content (Wang et al., 2024). Together, these findings support a systems-level approach to breeding for LDR, where the ideotype is achieved through the integration of multiple genetic and regulatory pathways rather than through single-gene modifications.

### 5.3 MicroRNAs and post-transcriptional regulation of LDR

The miRNAs regulate stem development and LDR through post-transcriptional mechanisms, primarily via mRNA cleavage or translational inhibition (Zhao et al., 2025). Several miRNA families have been implicated in traits directly relevant to culm mechanical strength and LDR. *miR156/miR529* targets the *SQUAMOSA PROMOTER BINDING PROTEIN LIKE* (*SPL*) gene family regulating tillering and plant height (Wang et al., 2015). By modulating tiller number and internode elongation, this miRNA family indirectly influences culm architecture and lodging susceptibility. *miR166* regulates HD-ZIP III affecting vascular bundle polarity and development (Zhang et al., 2018); given that vascular bundle number and arrangement are correlated with culm mechanical strength (Section 2.2), this miRNA may contribute to LDR through vascular architecture. *miR319* targets TCP transcription factors which regulate stem thickness and cell wall thickness (Fang et al., 2021), directly impacting culm mechanical properties. *miR444a* targets *OsMADS57*, which interacts with OsTB1 to repress *D14*, (the strigolactone receptor), thereby modifying plant architecture, including tillering and culm development (Guo et al., 2013).

Importantly, several of these miRNA-target modules intersect with the central DELLA-OsGSK2-BZR1 hormonal hub. For instance, the *miR156-SPL* module interacts with GA signaling pathways to regulate plant height (Wang et al., 2015), while the miR444a-OsMADS57-OsTB1 axis is directly connected to strigolactone signaling via *D14* (Guo et al., 2013), linking miRNA-mediated regulation to the broader hormonal network that controls culm architecture. Additionally, miR319-targeted TCP transcription factors have been shown to interact with BR signaling components, suggesting a role in integrating miRNA-mediated regulation with BR-dependent cell wall biosynthesis (Fang et al., 2021).

While direct evidence linking specific miRNAs to LDR phenotypes remains limited, the cumulative evidence indicates that miRNAs represent an important layer of post-transcriptional regulation that modulates culm architecture, vascular development, and cell wall properties. Future research should focus on characterizing the direct contributions of miRNA-target modules to LDR and their integration with the DELLA-OsGSK2-BZR1 module.

### 5.4 Epigenetic influences on stem development

Epigenetic mechanisms regulate gene expression without altering DNA sequences and play crucial roles in stem development (Pikaard and Mittelsten Scheid, 2014). Emerging evidence suggests that three distinct mechanisms may influence culm strength (**Figures 5A–C**).

**Figure 5:**
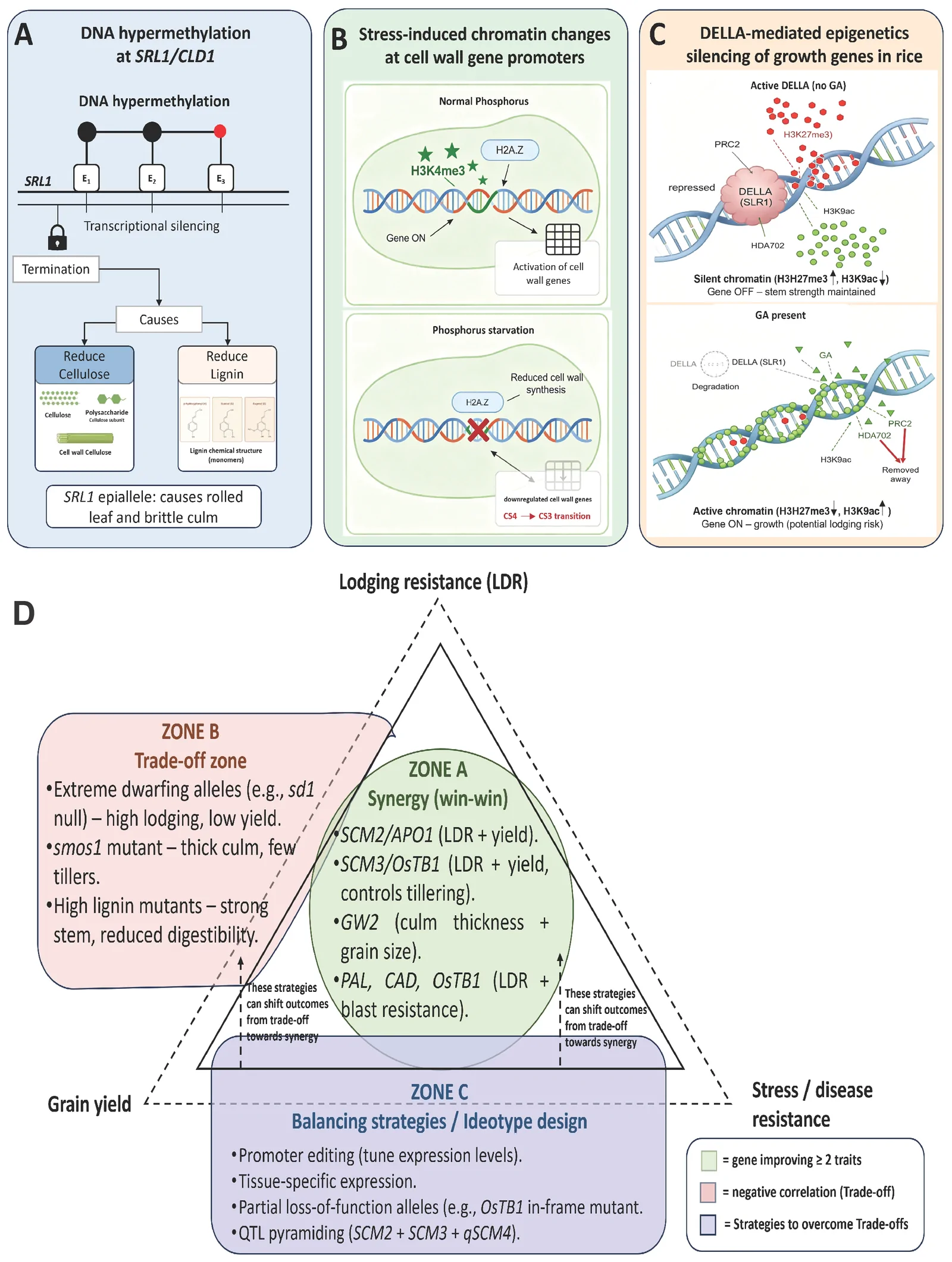
Epigenetic regulation, trait trade-offs, and the integrated model for LDR in rice. **(A)** DNA hypermethylation at *SRL1/CLD1*: hypermethylation of exon 3 (red circles) transcriptionally silences *SRL1* (termination), leading to reduced cellulose and lignin content. The *cld1* epiallele exhibits a rolled leaf and brittle culm phenotype. **(B)** Stress-induced chromatin changes at cell wall gene promoters: under normal phosphorus conditions, promoters of cell wall genes carry active H3K4me3 marks and H2A.Z variant, supporting normal expression and normal culm strength. Under phosphorus starvation causes loss of H3K4me3 while retaining H2A.Z, resulting in reduced gene expression and weakened culm. **(C)** DELLA-mediated epigenetic silencing (GA signaling): the DELLA protein SLR1 recruits the polycomb repressive complex 2 (PRC2) and histone deacetylase HDA702. PRC2 deposits H3K27me3 (repressive histone mark, red hexagonal), while HDA702 removes acetyl groups from H3K9 (green circle), together repressing cell wall and growth-related genes. GA (green triangle) signaling degrades DELLA, relieving this repression. Loss of repression leads to weak culm and reduced LDR. **(D)** Trade-offs and synergies among LDR, grain yield, and stress/disease resistance in rice.

First, the spontaneously arising epiallele *curled leaf dwarf 1* (*cld1*) allelic to *SEMI-ROLLED LEAF1* (*SRL1*) demonstrates that DNA hypermethylation can silence individual genes. Elevated methylation in exon 3 transcriptionally silences SRL1, reducing cellulose and lignin content. Treatment with 5-aza-dC partially restores expression, directly demonstrating that DNA methylation regulates cell wall synthesis (**Figure 5A**) (Li et al., 2017). While this study establishes a link between DNA methylation and cell wall composition, direct measurements of LDR in the *cld1* mutant have not been reported, and the contribution of this epigenetic mechanism to LDR remains to be determined.

Second, phosphorus starvation triggers chromatin changes within 24 hours; genes losing H3K4me3 while retaining H2A.Z are enriched in “plant-type cell wall” categories (pectinases, expansins), showing downregulated expression (**Figure 5B**) (Foroozani et al., 2020). This study demonstrates stress-induced chromatin remodeling at cell wall genes, but the direct impact on culm mechanical strength or LDR has not been established.

Third, DELLA protein SLR1 recruits PRC2 and HDA702 to maintain H3K27me3 and H3K9 deacetylation at target genes. Loss-of-function mutations reduce genome-wide H3K27me3 levels and derepress the expression of growth-related genes (**Figure 5C**) (Li et al., 2023). This finding provides a direct mechanistic link between GA signaling (via DELLA) and chromatin modification, suggesting that epigenetic plasticity may enables rice to adjust its mechanical properties in response to environmental changes. However, direct evidence linking this DELLA-mediated epigenetic mechanism to LDR phenotypes is currently lacking.

The epigenetic mechanisms described above are not isolated from the central DELLA-OsGSK2-BZR1 module or the NAC-MYB transcriptional cascade. Rather, they form an interconnected regulatory network that integrates environmental signals with developmental programs. The DELLA protein (SLR1), a core component of the GA signaling pathway, directly recruits Polycomb Repressive Complex 2 (PRC2) and histone deacetylase HDA702 to deposit repressive H3K27me3 marks and remove H3K9 acetylation at target genes, including those encoding cell wall biosynthetic enzymes and growth regulators (Li et al., 2023). This DELLA-mediated epigenetic silencing thus provides a direct mechanistic link between hormonal signaling (GA) and chromatin modification, enabling rapid and heritable transcriptional responses to environmental cues. Similarly, DNA methylation at loci such as *SRL1/CLD1* can silence genes involved in cell wall formation (Li et al., 2017), while stress-induced chromatin remodeling (e.g., loss of H3K4me3 at cell wall gene promoters under phosphorus starvation) modulates the accessibility of transcriptional regulators, including NAC and MYB transcription factors (Foroozani et al., 2020).

Collectively, these findings suggest that epigenetic modifications may represent an emerging regulatory layer that could impinge upon the DELLA–OsGSK2–BZR1 module and the NAC-MYB cascade, potentially integrating hormonal, developmental, and environmental signals to modulate culm strength. However, it is important to emphasize that direct evidence linking these epigenetic mechanisms to LDR remains limited. Most of the cited studies demonstrate effects on cell wall composition or gene expression rather than direct LDR measurements. Future research should prioritize the direct assessment of LDR phenotypes in epigenetic mutants to establish causal relationships between epigenetic regulation and LDR.

Recent studies have further expanded the understanding of epigenetic regulation in rice. Transposable element (TE)-mediated DNA methylation of the *NAC20* and *NAC26* promoters has been shown to regulate grain filling with a maternal effect in japonica rice, linking TE-mediated DNA methylation to NAC transcription factors and endosperm development (Wu et al., 2026). Multi-omics integration has mapped CHH methylation and gene regulatory networks across heat, drought, and salt stress in rice, revealing stress-specific chromatin dynamics and providing valuable resources for climate-resilient breeding (Guo et al., 2026). The identification of lysine β-hydroxybutyrylation (Kbhb) as a novel drought-responsive epigenetic mark further demonstrates the diversity of epigenetic modifications that can influence plant stress responses, with HDA710 identified as a key regulator of histone Kbhb in rice (Wei et al., 2026). These recent findings reinforce the notion that epigenetic modifications act as a regulatory layer, consistent with the integrated model discussed above (**Figure 6B**).

**Figure 6:**
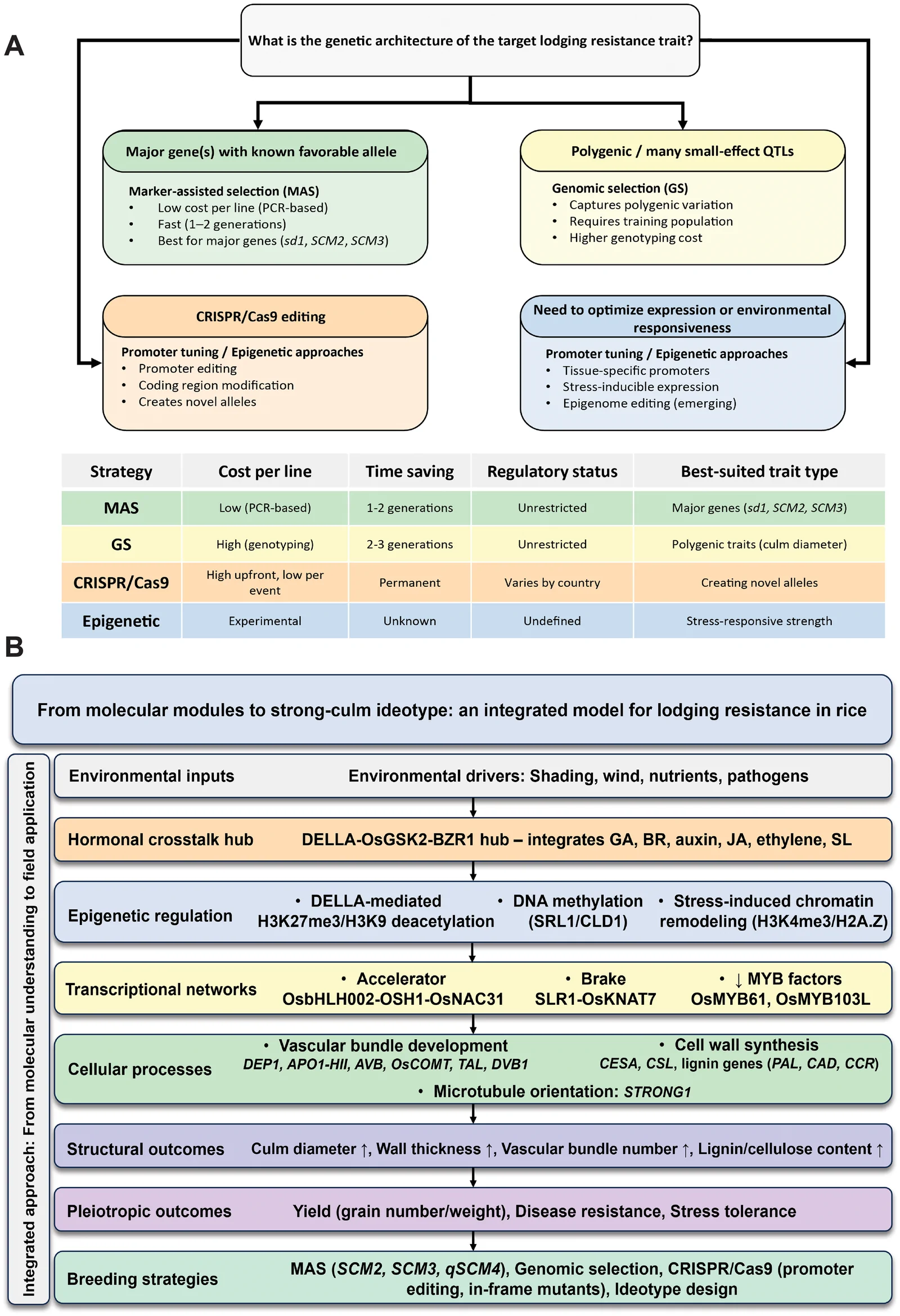
Breeding strategy decision tree and integrated model for LDR in rice. **(A)** Decision tree for selecting breeding strategies (MAS, GS, CRISPR/Cas9, promoter tuning/epigenetic approaches) based on trait genetic architecture. The table compares strategies by cost, time, regulatory status, and suitability. **(B)** Integrated multi-level model from environmental inputs to breeding strategies, incorporating hormonal crosstalk (DELLA-OsGSK2-BZR1), epigenetic regulation, transcriptional networks (accelerator/brake modules, MYB factors), cellular processes (vascular development, cell wall synthesis, microtubule orientation), structural outcomes, pleiotropic outcomes, and breeding applications (MAS, GS, CRISPR/Cas9, ideotype design). Downward arrows indicate the flow from molecular understanding to field application.

### 5.5 Synergy and trade-off mechanism between LDR, yield, and stress resistance

A major biological consideration in breeding for LDR is the potential negative correlation between LDR and yield traits, which arises from competitive resource allocation (**Figures 5D**, **6A**). However, it is important to emphasize that this correlation is not universal; rather, it depends on the specific genetic background, the traits being measured, and the environmental conditions under which plants are grown. Trade-offs are therefore genotype-specific (varying among different rice varieties), trait-specific (differing between traits such as culm diameter, plant height, and grain number), and environment-specific (influenced by growing conditions such as water availability, nutrient status, and weather).

Importantly, pleiotropic genes can overcome these trade-offs. For example, *SCM2/APO1* simultaneously increases stem diameter and grain number per panicle, breaking the negative correlation in specific genetic backgrounds (Ookawa et al., 2010a). Lignin biosynthesis genes (*PAL* and *CAD*) enhance both culm strength and blast resistance, as OsTB1 directly activates lignin biosynthesis while improving resistance to *Magnaporthe oryzae* (Zhao and Dixon, 2011; Tan et al., 2026). The *Ostb1* in-frame mutant enhances tillering and yield specifically under phosphorus deficiency without weakening culm strength, illustrating how environmental context can modulate trade-offs (Ishizaki et al., 2023). Strategies to mitigate these trade-offs include promoter editing, tissue-specific expression, and QTL pyramiding (**Figures 5D**, **6A**). The identification and deployment of “win-win” loci such as *SCM2/APO1* and *OsTB1* alleles that simultaneously improve multiple traits represent a promising approach for overcoming the classical yield-lodging trade-off.

## 6 Discussion and future perspectives

The ultimate goal of analyzing the molecular mechanisms underlying LDR is to apply them in breeding programs and to develop new rice varieties with high and stable yields. This section summarizes current breeding strategies, from gene discovery to variety development, and discusses how emerging biotechnologies can enable the precise improvement of LDR traits in rice. Quantitative targets, breeding population design, cost-effectiveness, phenotyping bottlenecks, and integration with hybrid rice are discussed.

### 6.1 Marker-assisted selection

Marker-assisted selection (MAS) enables selection of target genotypes at the seedling stage. Major effect genes (*sd1*, *d2*, *d61*) have been developed into molecular markers for hybrid rice improvement (Ashikari et al., 1999; Hong et al., 2003). Pyramiding additive QTLs (*SCM2*, *SCM3*, *qSCM4*) creates materials with thicker culms and enhanced LDR (Yano et al., 2015; Yang et al., 2023).

However, the successful application of MAS for LDR requires careful consideration of marker transferability and genetic-background effects. QTL effects are often context-dependent, with their expression and magnitude varying across different genetic backgrounds and environments (Kashiwagi et al., 2008; Chigira et al., 2023). For example, a QTL that confers a significant effect in one mapping population may show reduced or no effect when introgressed into a different genetic background, due to epistatic interactions or differences in the genetic architecture of the recipient line. Therefore, validation of markers in the target breeding population is essential before large-scale MAS implementation.

Kompetitive allele-specific PCR (KASP) markers have emerged as a cost-effective and high-throughput tool for MAS in rice, enabling large-scale genotyping with high accuracy and reproducibility. KASP-based markers have been successfully developed and validated for traits improving grain yield and adaptability of rice under direct-seeded cultivation conditions, demonstrating their utility in breeding programs (Sandhu et al., 2022). For LDR, KASP markers targeting validated QTLs such as *SCM2*, *SCM3*, and *qSCM4* could be developed and deployed to facilitate marker-assisted pyramiding of favorable alleles, provided they are validated in the target genetic background.

### 6.2 Designing the ideal culm architecture

An ideal plant type for LDR should possess a combination of morphological and mechanical traits that enhance culm strength. Based on studies in specific genetic backgrounds and environments, reported ranges for key traits include (1): a suitable height (130-150 cm for long-culm types) (2), robust lower internodes with a basal diameter greater than 4.0 mm (3), large section modulus (> 30 mm^3^) (4), high bending stress (> 800 gf mm^-2^) (5), wall-to-diameter ratio of approximately 0.2-0.3, and (6) lignin content 15-20% (Ookawa et al., 2010a; Kashiwagi, 2022; Mullangie et al., 2024). It is important to note that these values are not universal standards; rather, they represent reported ranges that may vary depending on the genetic background and environmental conditions. The optimal values for a given breeding program should be determined empirically, considering the specific genotype and target environment.

### 6.3 Breeding population and genomic selection

Biparental populations (F_2_, RILs) are classical for major QTL mapping; however, MAGIC populations capture greater allelic diversity, and backcross populations introgress QTLs into elite lines. Genomic selection (GS) uses high-density markers to predict performance without extensive phenotyping (Meuwissen et al., 2001). Heritability of culm traits is moderately high (0.6-0.8) in some genetic backgrounds, suggesting that GS could be effective for LDR breeding. Recent studies have directly evaluated GS for lodging-related traits in rice. For example, Lu et al. (2026) assessed GS for seven lodging-related traits in 438 rice accessions and reported prediction accuracies (Pearson’s r) ranging from 0.33 to 0.78 in cross-validation and 0.28 to 0.75 in independent prediction using the GBLUP model. Bending resistance exhibited lower prediction accuracy due to its lower genomic heritability, whereas indirect selection for highly heritable proxy traits (e.g., internode length and IL/PH ratio) was significantly more effective at identifying lodging-resistant cultivars. The study further demonstrated that GS can enrich the proportion of highly lodging-resistant accessions from 31.40% to 83.00% among selected individuals. Integration of GS with speed breeding (4–6 generations/year) can reduce the breeding cycle from 5–7 years to 2–3 years (Watson et al., 2018). However, GS for LDR remains at the research stage, with further validation needed across diverse genetic backgrounds and environments (Crossa et al., 2017).

### 6.4 CRISPR/Cas9 precision engineering

Gene editing enables precise modification of LDR. Demonstrated examples of successful CRISPR/Cas9 editing for LDR include: Editing *SLR1* generates new semi-dwarf alleles (*slr1-d7, slr1-d8*) with reduced height and unchanged yield (Jung et al., 2020). Promoter editing of *SCM3/OsTB1* produces alleles with thicker culms and larger panicles (Cui et al., 2020). A 30-bp in-frame deletion in *OsTB1* removed 10 amino acids between TCP and R domains, partially retaining protein function and increasing tiller number by ~40% without severe trade-offs, with a 38% yield increase observed specifically under phosphorus deficiency (Ishizaki et al., 2023).

Beyond these architectural modifications, cell wall engineering through CRISPR/Cas9 has emerged as a powerful strategy to directly enhance culm mechanical strength. Several successful examples illustrate this approach. First, the *MYB61–STRONG2* module represents a direct cell wall engineering target: *STRONG2* (encoding the mannan synthase CSLA5) is directly activated by MYB61, and its overexpression increases culm diameter and LDR by enhancing cell wall components such as mannose and cellulose, thereby improving sclerenchyma development in stems (Zhao et al., 2025b; Zhao et al., 2025a). Second, CRISPR/Cas9 editing of *DEP1* (encoding a G protein γ subunit) significantly enhances stem mechanical properties and LDR by increasing cellulose and hemicellulose contents while reducing lignin content and cellulose crystallinity (Wang et al., 2024). Third, CRISPR/Cas9-induced mutations in *OsIRX10* (the *FC19* locus) reduce hemicellulose and cellulose contents but increase lignin and arabinose levels; despite the resulting brittleness, these mutants display increased breaking force and improved LDR, demonstrating that cell wall remodeling can simultaneously enhance mechanical strength and biomass saccharification (Dang et al., 2023).

The composition of lignin monomers also plays a critical role in culm mechanical properties. Lignin is composed of three major monolignols: p-hydroxyphenyl (H), guaiacyl (G), and syringyl (S) units. The S/G ratio influences cell wall rigidity and degradability, with evidence suggesting that G, S, and H monomers may differentially contribute to cell wall strength and LDR (Liu et al., 2018). While the ideal S/G ratio for optimal culm strength remains to be fully defined, modulating lignin monomer composition through targeted engineering of lignin biosynthetic genes (e.g., *OsCOMT*, *OsF5H*) offers a promising avenue for tailoring cell wall properties to enhance mechanical strength without compromising other agronomic traits.

In addition to these demonstrated examples, several candidate targets have been proposed for cell wall engineering to enhance LDR, though they require further validation through successful editing and phenotypic characterization. These candidate targets include *OsMYB61*, *OsMYB103L*, and *OsCESA* genes, which regulate secondary wall biosynthesis and cellulose deposition (Yang et al., 2014; Zhao et al., 2025b). *OsMYB61* directly activates *STRONG2* expression and has been validated through overexpression and knockout studies; however, direct editing of *OsMYB61* specifically for LDR has not yet been demonstrated and remains a promising target for future research. Similarly, *OsCESA* genes are well-established regulators of cellulose synthesis, but their targeted editing for LDR requires further investigation.

### 6.5 Cutting-edge technologies

Emerging technologies are increasingly being applied to study culm structure and LDR, though their direct application to lodging-related traits varies. High-throughput phenotyping using x-ray micro-CT and RGB imaging enables non-destructive measurements of culm traits such as internode diameter, wall thickness, and vascular bundle arrangement, with direct relevance to LDR assessment (Wu et al., 2019). single-cell transcriptomics has been applied to reveal cell heterogeneity during stem development (Wang et al., 2025); while this technology holds promise for understanding cell-type-specific responses relevant to culm strength, its direct application to LDR has not yet been demonstrated and remains an area for future investigation. Confocal Raman microscopy enables high-resolution visualization of lignin, cellulose, and hemicellulose distribution in the cell walls (Huang et al., 2025), providing insights into the spatial organization of wall polymers that influence mechanical properties. These technologies drive LDR research toward functional mechanism analysis and precision breeding.

Field-scale phenotyping feasibility and cost remain significant challenges for the widespread adoption of these technologies. While x-ray micro-CT and confocal Raman microscopy offer high-resolution data, they are currently limited to laboratory settings due to high instrument costs, low throughput, and the need for sample preparation. RGB imaging-based phenotyping platforms are more scalable and cost-effective, but their ability to capture detailed internal culm traits is limited. The application of single-cell transcriptomics at field scale is currently impractical due to high costs and the complexity of sample preparation and data analysis. Future efforts should focus on developing cost-effective, high-throughput field phenotyping platforms that can integrate multiple imaging modalities, as well as on establishing the relationship between laboratory-measured traits and field LDR. These technologies drive LDR research toward functional mechanism analysis and precision breeding, but their translation to routine breeding programs will require continued technological refinement and cost reduction (Wu et al., 2019).

### 6.6 Practical considerations

MAS is most cost-effective for introgressing known major QTLs; GS requires substantial training population investment; CRISPR offers precision but faces regulatory heterogeneity (**Table 3**; **Figure 6A**). Current regulatory frameworks for gene-edited crops vary considerably across major jurisdictions:

**Table 3:** Cost-effectiveness comparison of breeding strategies for lodging resistance.

Method	Cost per line	Time saving	Best suited for	Key reference
Conventional phenotyping	Low (field space, labor)	Baseline	Initial screening, training populations	–
MAS (few loci)	Low ~$3.82 per sample (KASP)	1-2 cycles	Major genes (*sd1*, *SCM2*, *SCM3*)	(Sandhu et al., 2022)
GS (genome-wide)	Moderate–High; ~$4.80–$40 per sample	2-3 cycles	Polygenic traits (culm diameter, wall thickness)	(Crossa et al., 2017)
CRISPR/Cas9 editing	High upfront, low per event	Heritable (once established)	Creating novel allelic variation	(Thakur et al., 2026)

In China, the Ministry of Agriculture and Rural Affairs (MARA) issued the Guidelines for Safety Evaluation of Gene-Edited Plants for Agricultural Use (Trial) in January 2022 (USDA Foreign Agricultural Service, 2022), followed by the Rules for Review of Gene-Edited Plants for Agricultural Use (Trial) in April 2023 (USDA Foreign Agricultural Service, 2023). These guidelines adopt a case-by-case, risk-based approach, classifying GE plants into four categories based on whether the target trait increases environmental and food safety risks. In 2024 and 2025, China granted safety certificates for gene-edited crops including soybeans, wheat, corn, and rice (Thakur et al., 2026). The simplified approval process is estimated to yield annual welfare gains of $52–57 billion USD from accelerated R&D transfer (Jin et al., 2025).

In the United States, the USDA-APHIS regulates gene-edited crops through a case-by-case determination of whether the modified organism is a “regulated article” under 7 CFR Part 340 (Thakur et al., 2026). Gene-edited crops that do not introduce foreign DNA and contain edits that could be achieved through conventional breeding are generally exempted from USDA regulation (Thakur et al., 2026).

In the European Union, a provisional agreement reached in December 2025 established a dual-track system for New Genomic Techniques (NGTs) (Salomón et al., 2026):

1. Category 1 NGT plants (equivalent to conventional breeding; changes limited to 20 nucleotide substitutions or insertions, no foreign DNA) are exempt from genetically modified organism (GMO) regulations and follow simplified verification procedures.
2. Category 2 NGT plants remain subject to the existing genetically modified organisms (GMO) regulatory framework (Salomón et al., 2026).

A combined approach using MAS for major genes and exploring GS for polygenic traits is currently the most pragmatic path, with CRISPR/Cas9 offering targeted precision for specific traits where regulatory pathways are clear (Crossa et al., 2017; Hickey et al., 2017).

### 6.7 Integrating LDR with stress resilience and yield

Breaking the negative correlation between LDR, yield, and stress resistance remains a key challenge. A systematic workflow for identifying and engineering “win-win” loci can be envisioned:

1. GWAS discovery: Multi-trait genome-wide association studies can identify genomic regions associated with both culm strength and stress tolerance.
2. Candidate validation: Functional validation through gene editing or transgenic approaches confirms the role of candidate genes in both traits.
3. Causal variant identification: Fine-mapping and promoter analysis identify specific polymorphisms or regulatory elements responsible for the pleiotropic effects.
4. Targeted engineering: Promoter editing or CRISPR/Cas9-mediated modification of causal variants can enhance both traits simultaneously.
5. Field validation: Multi-environment trials assess the stability of improved traits across diverse conditions.

This workflow is exemplified by the identification of *OsCam3* through multi-omics integration as a key regulator of heat stress responses, with promoter editing representing a potential intervention point for enhancing both stress tolerance and culm strength (Guo et al., 2026). Silicon and potassium applications enhance both stem strength and abiotic stress tolerance (Hu et al., 2022; Mullangie et al., 2024). Identifying hub genes that simultaneously regulate cell wall strength and stress responses is crucial for synergistic improvement.

Recent multi-omics studies have provided valuable resources for integrating stress resilience with yield improvement. Multi-omics integration has mapped CHH methylation and gene regulatory networks across heat, drought, and salt stress in rice, identifying key hub genes such as *OsCam3* that regulate heat stress responses through epigenetic and transcriptional mechanisms (Guo et al., 2026). These findings provide a framework for identifying additional ‘win-win’ loci that simultaneously regulate cell wall strength and stress responses.

### 6.8 Epigenetic breeding

Epigenetic variation regulates cell wall synthesis (Section 5.4). The *cld1* epiallele demonstrates that hypermethylation silences *SRL1* and reduces cellulose/lignin. However, several challenges must be addressed before epigenetic breeding can be practically deployed:

1. Stability: The heritability of epigenetic marks across generations remains uncertain.
2. Heritability: Whether epigenetic modifications are faithfully transmitted to progeny is not well established.
3. Specificity: Current approaches lack the precision to target specific loci without off-target effects.

Although no practical epigenome editing tools have been deployed in rice, targeting such loci with chemical demethylating agents or synthetic modifiers could dynamically regulate cell wall strength in response to stress. Recent high-impact studies have provided proof-of-concept for the potential of epigenetic engineering in rice. Transposable element-mediated DNA methylation of the NAC20 and NAC26 promoters has been shown to regulate grain filling with a maternal effect in japonica rice, with deletions of these TEs by gene editing leading to decreased methylation and biallelic expression (Wu et al., 2026). This provides proof-of-concept that targeted removal of TE-mediated methylation can alter gene expression patterns. Multi-omics integration has mapped CHH methylation and gene regulatory networks across heat, drought, and salt stress in rice, providing valuable resources for climate-resilient breeding (Guo et al., 2026). The discovery of lysine β-hydroxybutyrylation (Kbhb) as a novel drought-responsive epigenetic mark in rice further expands the repertoire of epigenetic modifications that could be targeted for breeding applications, with HDA710 identified as a key regulator of histone Kbhb during drought stress (Wei et al., 2026).

Despite these promising findings, epigenetic breeding remains a preliminary and emerging field. The stability, heritability, and specificity of epigenetic modifications require further investigation before they can be reliably deployed in breeding programs. As outlined in the integrated model (**Figure 6B**), epigenetic modifications act as a regulatory layer that impinges upon the DELLA-OsGSK2-BZR1 module and the NAC-MYB cascade, but direct evidence linking epigenetic modifications to improved LDR phenotypes is currently lacking. Future research should prioritize the direct assessment of LDR phenotypes in epigenetic mutants and the characterization of epigenetic marks at lodging-related QTLs to establish causal relationships between epigenetic regulation and LDR.

### 6.9 Synthesis and broader implications

The integrated model presented in this review (**Figure 6B**) reveals that LDR is not a single trait but rather the emergent property of a multi-level regulatory network spanning hormonal signaling, transcriptional cascades, epigenetic regulation, and structural cell wall deposition. This synthesis has several broader implications. First, it suggests that breeding for LDR should adopt a systems approach rather than targeting individual traits in isolation. Second, the identification of hub genes such as *SCM2/APO1* and *OsTB1* that simultaneously regulate multiple traits offers a path to overcome classical trade-offs. Third, the emerging role of epigenetic regulation highlights the need to consider both genetic and environmental influences on culm strength. Finally, the convergence of mechanistic understanding with advanced breeding technologies (CRISPR, GS, speed breeding) positions the field for accelerated progress in developing climate-resilient rice varieties.

The challenge ahead lies not in identifying individual genes, but in understanding how these components interact across scales, from molecules to field-level phenotypes, to produce the integrated phenotype of LDR. While our model integrates molecular and physiological processes, it is important to acknowledge that genotype-by-environment (G×E) interactions and ecosystem-level factors such as: soil properties, weather patterns, and biotic stress pressures, also play critical roles in determining lodging susceptibility. These factors are discussed in Section 1 (environmental factors) and Section 2.3 (agronomic and environmental factors), but systematic integration of G×E interactions into the molecular framework remains an important area for future research.

## 7 Concluding remarks

This review provides a comprehensive synthesis of the molecular mechanisms underlying culm-associated LDR in rice, from hormonal crosstalk to cell wall engineering to breeding applications. Nine priority areas for future research are as follows:

A. Systematically mine superior alleles from wild rice and landraces using pan-genome approaches.
B. Elucidate how *SCM2/APO1* achieves synergistic LDR and yield improvement at single-cell resolution.
C. Develop multi-omics predictive models that incorporate genotype-by-environment interactions.
D. Harmonize regulatory frameworks for gene-edited crops to accelerate deployment.
E. Exploring root-culm synergy mechanisms for direct-seeded rice system.
F. Investigating whether epigenetic marks at cell wall loci can be stably inherited for breeding applications.
G. Establish standardized lodging phenotyping protocols to enable reliable comparisons across studies and environments.
H. Conduct multi-environment validation of candidate genes and QTLs to assess stability and transferability across diverse genetic backgrounds and growing conditions.
I. Directly test the proposed DELLA-OsGSK2-BZR1 → cell wall → mechanical strength pathway through targeted gene editing and comprehensive mechanical phenotyping.

With the ongoing integration of mechanistic understanding and advanced breeding technologies, the development of high-yielding, lodging-resistant rice varieties is an achievable goal that will contribute to global food security. This review bridges the DELLA-OsGSK2-BZR1 module to cell wall biosynthesis with emerging and promising breeding strategies, offering a roadmap for designing next-generation lodging-resistant rice varieties (**Figure 6B**).
